# Supplementary material for: Electrochemical oxo-functionalization of cyclic alkanes and alkenes using nitrate and oxygen
Source: Nat Commun. 2023 Jul 28;14:4565. doi: 10.1038/s41467-023-40259-0 (PMC10382549; doi:10.1038/s41467-023-40259-0)
Supplement: Supplementary file 1 — Supplementary Information [file 41467_2023_40259_MOESM1_ESM.pdf]

Supplementary Information

Electrochemical oxo-functionalization of cyclic alkanes and alkenes using nitrate and oxygen

Joachim Nikl[1], Kamil Hofman[1], Samuel Mossazghi[1], Isabel C. Möller[1], Daniel Mondeshki[1], Frank Weinelt[2], Franz-Erich Baumann[2] & Siegfried R. Waldvogel*[1]

[1]Department of Chemistry, Johannes Gutenberg University Mainz, Duesbergweg 10–14, 55128 Mainz, Germany

[2] Evonik Operations GmbH, Paul-Baumann-Straße 1, 45772 Marl, Germany

*Correspondence to: waldvogel@uni-mainz.de

Content

Content 2
1. Supplementary Methods 3
  1.1 General information 3
  1.2 Supplementary set-up information for the general protocols (GP) 5
    1.2.1 Set-up to GP 1: 5 mL PTFE cell 5
    1.2.2 Set-up to GP 2: 100 mL three-necked round-bottom flask 5
    1.2.3 Set-up to GP 3: 25 mL beaker-type cell 6
    1.2.4 Set-up to GP 4: Electrochemical flow cell (2 x 6 cm^2) 6
    1.2.5 Set-up for oxygen supply 7
2. Supplementary Results 8
  2.1 External GC calibration with an internal standard for yield determination 8
  2.2 Optimization for cycloalkane oxidation 10
  2.3 Optimization for cycloalkene oxidation 11
  2.4 Co-electrolysis reactions for cyclooctane (1c) and cyclooctene (5b) 12
  2.5 Optimization for cycloalkene oxidation in flow 12
  2.6 Optimization for benzylic oxidation 13
  2.7 Recovery and reuse of the supporting electrolyte 15
  2.8 Oxo-functionalization results with branched cycloalkanes 16
  2.9 Qualitative HRMS analysis of the reaction mixture for diacid synthesis 19
3. Supplementary Notes 20
  3.1 Determination of dissolved oxygen concentration 20
  3.2 Mechanism elucidation experiments 23
    3.2.1 Griess test 23
    3.2.2 Ion chromatography 23
    3.2.3 pH test 23
    3.2.4 Peroxide test with titanyl sulfate 24
    3.2.5 Karl Fischer titration 24
    3.2.6 Control experiment for nitrate radical observation 25
    3.2.7 Cyclic voltammetry studies 26
  3.3 *N*-Acetylbenzamide formation 26
  3.4 Syntheses of supporting electrolytes 27
  3.5 Characterization of oxo-functionalization products 28
4. Supplementary Spectra (literature unreported) 35
5. Supplementary References 46

1. Supplementary Methods

1.1 General information

Chemicals were of analytical grade and were obtained from common chemical providers such as TCI, Aldrich, Fluka, and Acros. Oxygen gas was purchased in technical quality of 2.5 from NIPPON GASES Germany GmbH, Düsseldorf, Germany, and used without purification.

As **electrode materials**, SIGRADUR® G glassy carbon, from HTW Hochtemperatur Werkstoffe GmbH, Thierhaupten, Germany, DIACHEM™ boron-doped diamond (BDD, 15 µm diamond layer on silicon support) from CONDIAS GmbH, Itzehoe, Germany and Sigrafin™ V2100 isostatic graphite from SGL Carbon, Bonn, Germany, were used.

As **electronic equipment**, a HMP4040 Programmable Power Supply 384 W, from Rohde & Schwarz GmbH & Co. KG, München, Germany, and a MR Hei-Tec magnetic stirrer, from Heidolph Instruments GmbH & Co.KG, Kelkheim, Germany, were used.

Column chromatography was performed on silica gel 60 M (0.040–0.063 mm, Macherey-Nagel GmbH & Co. KG, Düren, Germany). Therefore, a preparative chromatography system (Büchi, Flawil, Switzerland) was used with a Büchi Control Unit C-620, an UV detector Büchi UV photometer C-635, a Büchi fraction collector C-660 and two Pump Modules C-605 for adjusting the solvent mixtures. As eluent, mixtures of cyclohexane and ethyl acetate (technical grade, purified via distillation prior to use) were used. Optionally, glacial acetic acid was used as an additive. Ratios of the solvent mixture refer to volume ratios.

Thin layer chromatography was performed with silica gel 60 sheets on aluminium (F254, Merck KGaA, Darmstadt, Germany). A potassium permanganate stain (3 g $KMnO_4$, 20 g K_2CO_3, 5 mL NaOH (5%), 300 mL water) and a *p*-anisaldehyde stain (135 mL EtOH, 5 mL conc. H_2SO_4, 1.5 mL of AcOH (100%), 3.7 mL *p*-anisaldehyde) were used for visualization of components.

High performance liquid chromatography was performed on a Shimadzu HPLC-MS with a SIL-20A HT autosampler, a CTO-20AC column oven, two LC-20AD pump modules for adjusting the eluent, a SPD-M20A photodiode array detector, a LCMS-2020 mass spectrometer, a CBM-20A system controller (all: Shimadzu, Japan) and a Eurosphere II 100-5 C18 column (150 x 4 mm, KNAUER Wissenschaftliche Geräte GmbH, Berlin, Germany). Eluent: acetonitrile/water (1:9 → 10:0) + formic acid (1 vol.%).

Gas chromatography for non-acidic compounds was performed on a Shimadzu GC-2025 (Shimadzu, Japan) using a HP-5MS column (Agilent Technologies, Santa Clara, California; length: 30 m, inner diameter: 0.25 mm, film: 0.25 µm, carrier gas: hydrogen). For acidic compounds a Shimadzu GC-2010 (Shimadzu, Japan) equipped with a Zebron ZB-FFAP column (Phenomenex Ltd., Aschaffenburg, Deutschland; length: 30 m, inner diameter: 0.25 mm, film: 0.25 µm, carrier gas: argon) was used. GC-MS measurements were carried out on a Shimadzu GC-2010 (Shimadzu, Japan) using a HP-1 column (Agilent Technologies, Santa Clara, California; length: 30 m, inner diameter: 0.25 mm, film: 0.25 µm, carrier gas: helium). The chromatograph was coupled to a mass spectrometer Shimadzu GC-MS-QP2010. For GC sample preparation a column filtratrion was performed over silica gel 60 M (0.040–0.063 mm, Macherey-Nagel GmbH & Co. KG, Düren, Germany).

Melting ranges (m_R) were determined with a Melting Point Apparatus B-565 (Büchi, Flawil, Switzerland) and are uncorrected. Heating rate: 1 °C min^{-1}.

NMR spectroscopy of ^1H, ^{13}C and ^{19}F spectra were recorded at 25 °C, using a Bruker Avance II 400 (400 MHz, 5 mm BBFO-SmartProbe with z gradient and ATM, SampleXPress 60 sample changer, Analytische Messtechnik, Karlsruhe, Germany). Chemical shifts (δ) are reported in parts per million (ppm) relative to traces of $CHCl_3$, DMSO-d_5 or HDO in the corresponding deuterated solvent. For ^{19}F spectra $CFCl_3$, for ^{31}P spectra H_3PO_4 and for ^{13}C NMR spectra Me_4Si are serving as reference compounds if not referenced to the used deuterated solvent[1]. On-line reaction monitoring was performed with a

Spinsolv 60 benchtop NMR spectrometer (60 MHz, resolution: <0.5 Hz (50%) for ^{1}H, Magritek GmbH, Aachen, Germany).

High-resolution mass spectra were obtained by using an Agilent 6545 QTOF-HRAM-MS (Agilent Technologies, Santa Clara, California) apparatus employing $ESI^{+/-}$ and $APCI^{+/-}$.

Cyclic voltammetry was performed in a 10 mL snap-cap vial equipped with an Autolab PGSTAT101 potentiostat (Metrohm AG, Herisau, Switzerland). WE: glassy carbon disk, 3 mm diameter (7.07 mm^2 area); CE: glassy carbon rod; RE: Ag/AgCl in saturated LiCl/EtOH. Solvent: acetonitrile. $v = 0.02–1.5\ V\ s^{-1}$, supporting electrolyte: NBu_4PF_6 or NBu_4NO_3 (0.1 mol L^{-1}).

Ion chromatography was performed on an 850 Professional IC (Metrohm AG, Herisau, Switzerland) equipped with a Metrosep A Supp 4 anion exchange column (Metrohm AG, Herisau, Switzerland). Eluent system: Na_2CO_3 (0.9 mmol L^{-1}) and $NaHCO_3$ (0.85 mmol L^{-1}) in H_2O with 5 vol.% acetone.

Coulometric Karl Fischer titration was performed on a Titrando 851 (Metrohm AG, Herisau, Switzerland) equipped with a Stirrer 801 and a Generator electrode with diaphragm (both: Metrohm AG, Herisau, Switzerland). Anolyte: Hydranal™ Coulomat AG (Honeywell, Morristown, USA), Catholyte: Hydranal™ Coulomat CG (Honeywell, Morristown, USA). Analysis program: tiamo™ (Metrohm AG, Herisau, Switzerland).

1.2 Supplementary set-up information for the general protocols (GP)

1.2.1 Set-up to GP 1: 5 mL PTFE cell

The electrolysis set-up is commercially available as IKA Screening System from IKA-Werke GmbH & Co. KG, Staufen, Germany.

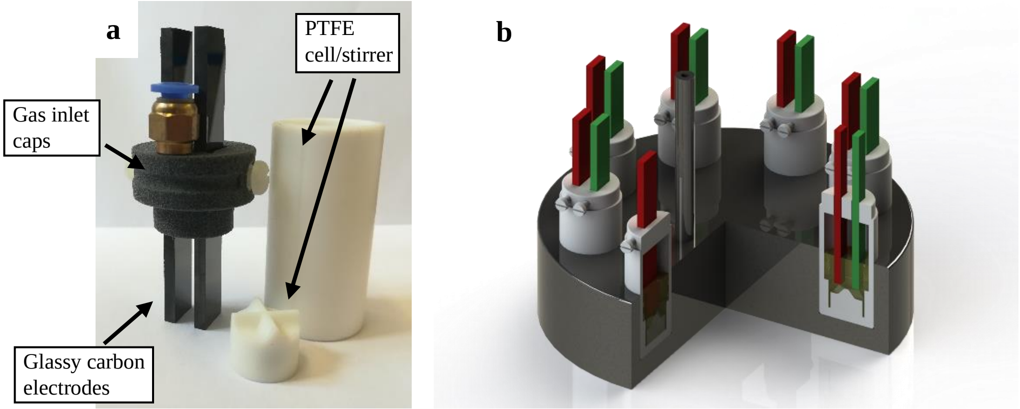

Supplementary Fig. 1: a Single-part 5 mL PTFE cell with glassy carbon electrodes. **b** Schematic representation of an 8-fold screening set-up.

1.2.2 Set-up to GP 2: 100 mL three-necked round-bottom flask

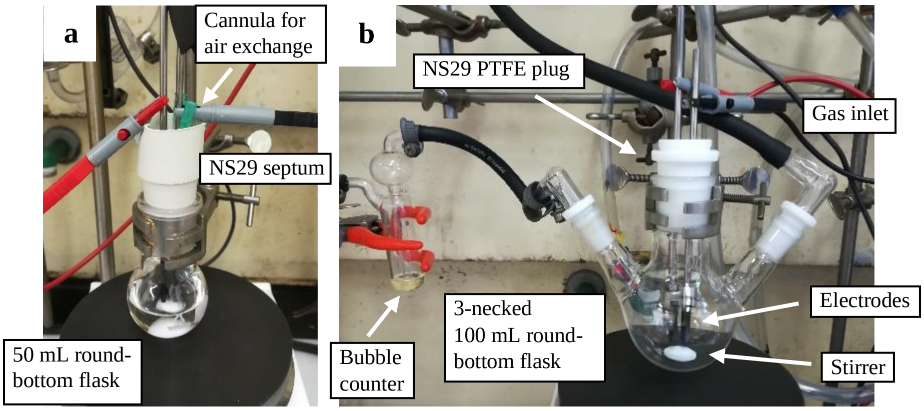

Supplementary Fig. 2: a 50 mL round-bottom flask for electrolysis with a NS29 septum including electrode holders, cannula for air exchange and a magnetic stirrer. **b** 100 mL three-necked round-bottom flask for electrolysis with a NS29 PFTE plug including electrode holders, a magnetic stirrer and a bubble counter.

1.2.3 Set-up to GP 3: 25 mL beaker-type cell

The cells are commercially available as SynLectro™ Electrolysis Platform from Sigma-Aldrich (Merck KGaA, Darmstadt, Germany).

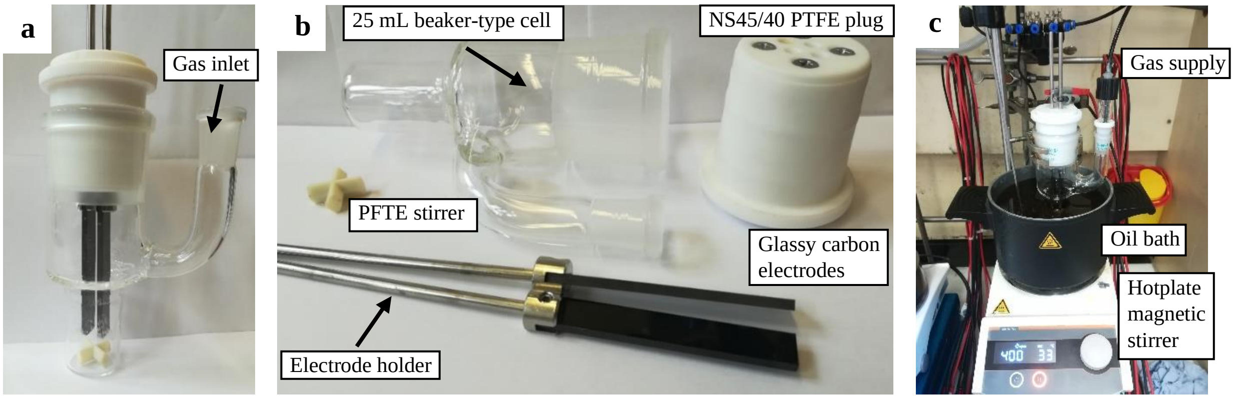

Supplementary Fig. 3: a Assembled 25 mL beaker-type glass cell with gas inlet attachment, manufactured according to specifications from the Waldvogel group by Hans W. Schmidt GmbH & Co. KG, HWS Labortechnik, Mainz, Germany. **b** Disassembled cell. **c** Set-up with gas supply and oil bath.

1.2.4 Set-up to GP 4: Electrochemical flow cell (2 x 6 cm^2)

The electrolysis set-up is commercially available as IKA ElectraSyn flow from IKA-Werke GmbH & Co. KG, Staufen, Germany.

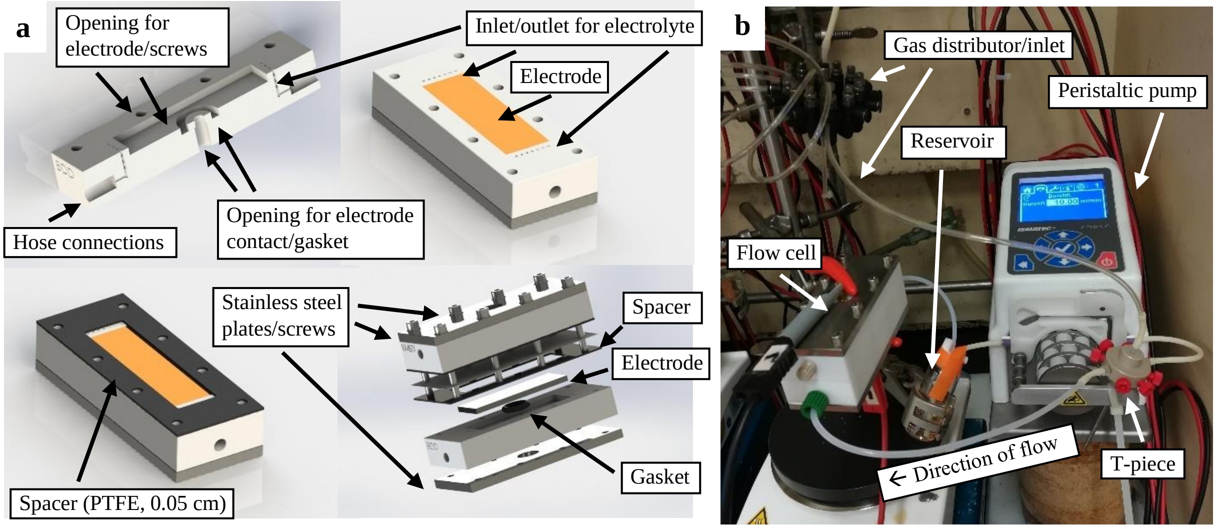

Supplementary Fig. 4: a Schematic disassembled flow cell. **b** Set-up for electrochemical reactions in flow including a peristaltic pump, a gas inlet, the flow cell and a reservoir.

1.2.5 Set-up for oxygen supply

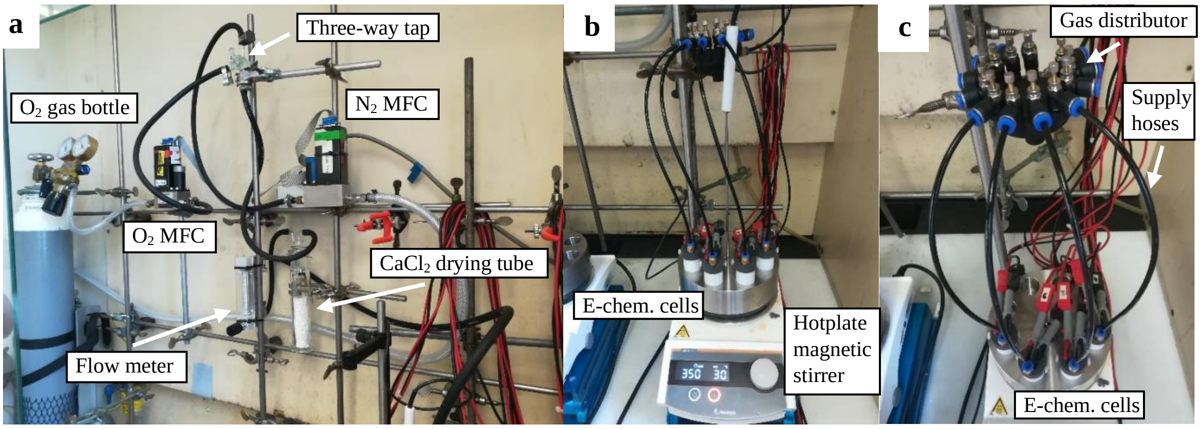

Supplementary Fig. 5: **a** Gas inlet apparatus. **b** Distributor connection to the electrolysis cells. **c** View from above onto the gas distributor.

The gas inlet was controlled via two 5850S mass flow controllers (MFC) from Brooks Instrument B.V., Veenendaal, Netherlands. One controller was used for the oxygen line and one for the nitrogen line. The controllers were operated using Smart DDE software and Matlab R2017b. The volume flow rate was additionally monitored using a DK800 flow meter from KROHNE Messtechnik GmbH, Duisburg, Germany. The total volume flow rate was a constant 20 mL min^{-1} for all tests carried out, which, limited by the MFCs used, also represents the maximum achievable volume flow rate. The percentage volume flows of the two gases were set using the MFCs and their software. The gases used were Oxygen 2.5 from NIPPON GASES Germany GmbH, Düsseldorf, Germany and Nitrogen 5.0 from NIPPON GASES Germany GmbH, Düsseldorf, Germany. The gas distributor as well as the gas inlet lids of the electrolysis cells were purchased from IKA-Werke GmbH & Co. KG, Staufen, Germany[2].

2. Supplementary Results

2.1 External GC calibration with an internal standard for yield determination

For the yield determination via gas chromatography, a calibration was carried out. 1,3,5-trimethoxybenzene (10 mg for each measurement) was used for ketone products and propionic acid (50.5 µL for each measurement) was used for carboxylic acids as internal standard. The molar ratio between the product and the standard *n*(P)/*n*(Std) was related to the measured GC integral ratios between product and standard GC-Int.(P)/GC-Int.(Std). From the resulting linear regression equation, the amount of product substance in an unknown sample can be calculated. The statistical software Origin 7.5 SR6 (OriginLab Corporation, Northampton, Massachusetts) was used for linear regression analysis. Mathematical calculations were performed using Microsoft® Excel® 2019.

Preparation of the calibration solutions: The analyte is dissolved together with the standard in the solvent (acetonitrile or isobutyronitrile, 5 mL) in different concentrations. Three drops form the solution are filtered over silica gel 60 M (approx. 330 mg) into a GC vial (eluent: ethyl acetate).

a

n(P) / *n*(Std)	GC int.(P) / GC int.(Std)
0.2 ± 0.1	0.203 ± 0.005
0.73 ± 0.06	0.89 ± 0.01
1.390 ± 0.005	1.69 ± 0.01
3.6 ± 0.2	4.46 ± 0.02
7.2 ± 0.6	9.1 ± 0.1

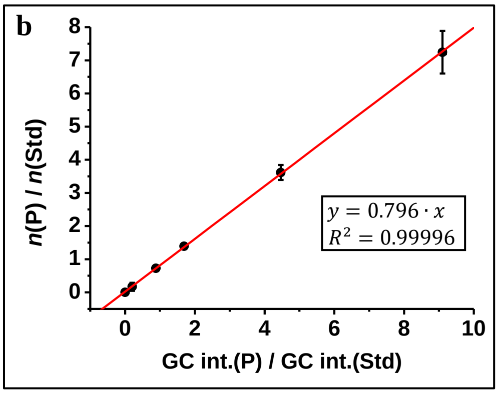

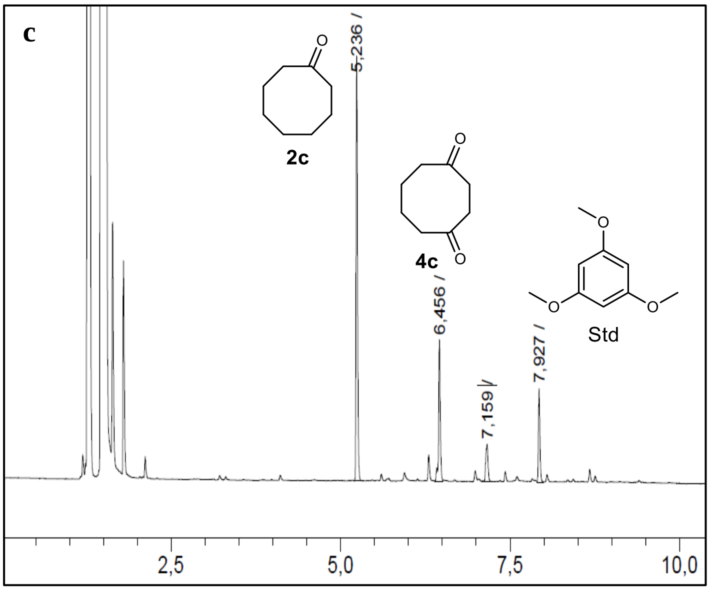

Supplementary Fig. 6: **a** Data of the calibration measurements for cyclooctanone (**2c**). Of each concentration, three samples were prepared and analyzed. **b** External GC calibration of cyclooctanone (**2c**) using 10 mg of 1,3,5-trimethoxybenzene as internal standard for each measurement. Error bars along the *y*-axis are calculated via uncertainty propagation, and error bars along the *x*-axis represent standard deviation with 3 independent replicates. Linear regression straight line is labeled in red. **c** Example GC chromatogram after an electrolysis to visualize the evaluation.

The calibration for the yield determination of cyclododecanone (**2e**) has been carried out analog to cyclododecanone (**2c**).

a

n(P) / n(Std)	GC int.(P) / GC int.(Std)
0.11 ± 0.01	0.332
0.220 ± 0.007	0.734
0.330 ± 0.005	1.118
0.440 ± 0.003	1.417

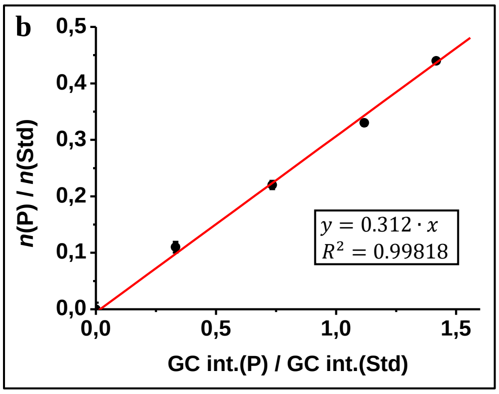

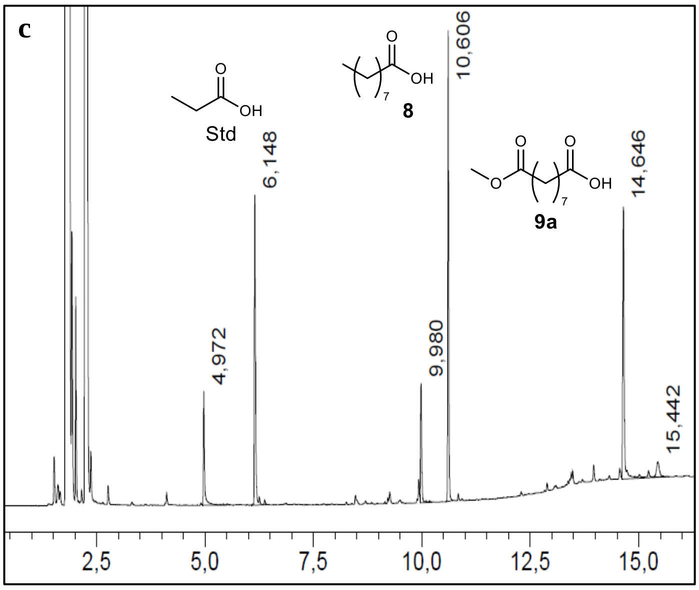

Supplementary Fig. 7: **a** Data of the calibration measurements for azelaic acid monomethyl ester (**9a**). **b** External GC calibration of azelaic acid monomethyl ester (**9a**) using 50.5 µL of propionic acid as internal standard for each measurement. Error bars along the *y*-axis are calculated via uncertainty propagation, and error bars along the *x*-axis represent standard deviation with 3 independent replicates. Linear regression straight line is labeled in red. **c** Example GC chromatogram after an electrolysis to visualize the evaluation.

The calibration for the yield determination of pelargonic acid (**8**) has been carried out analog to azelaic acid monomethyl ester (**9a**).

2.2 Optimization for cycloalkane oxidation

Supplementary Table 1: Optimization reactions for cyclooctane (**1c**) oxidation, according to GP 1.

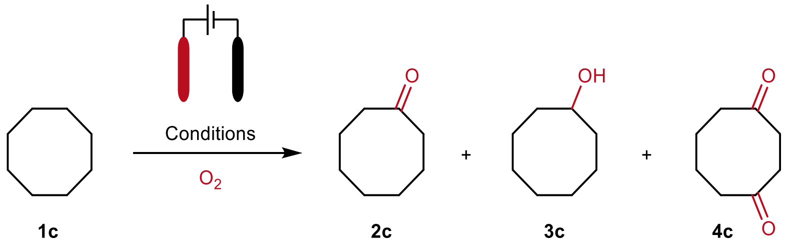

Entry	Deviation from standard conditions[a]	2c[b]	3c[c]	4c[c]
1	None	16%	1%	1%
2	6 *F*	16%	1%	1%
3	8 *F*	15%	1%	1%
4	5 mA cm^{-2}	7%	1%	0%
5	20 mA cm^{-2}	19%	1%	1%
6	30 mA cm^{-2}	18%	2%	1%
7	60 mA cm^{-2}	9%	8%	0%
8	20 mA cm^{-2}, O_2/N_2 = 0/100	0%	0%	0%
9	20 mA cm^{-2}, O_2/N_2 = 20/80	5%	5%	0%
10	O_2/N_2 = 5/95	6%	3%	0%
11	O_2/N_2 = 10/90	7%	3%	0%
12	O_2/N_2 = 20/80	31%	1%	6%
13	Air ($O_2/N_2 \approx 21/78$)	23%	2%	3%
14	O_2/N_2 = 35/65	9%	2%	0%
15	O_2/N_2 = 50/50	15%	1%	1%
16	O_2/N_2 = 20/80, **1c** (0.1 mol L^{-1}), NBu_4NO_3 (0.5 eq.)	30%	2%	4%
17	O_2/N_2 = 20/80, **1c** (0.1 mol L^{-1}), NBu_4NO_3 (1.0 eq.)	30%	1%	6%
18	O_2/N_2 = 20/80, **1c** (0.2 mol L^{-1}), NBu_4NO_3 (0.2 eq.)	26%	2%	4%
19	O_2/N_2 = 20/80, **1c** (0.2 mol L^{-1}), NBu_4NO_3 (1.0 eq.)	27%	1%	4%
20	O_2/N_2 = 20/80, **1c** (0.5 mol L^{-1}), NBu_4NO_3 (0.2 eq.)	23%	1%	4%
21	O_2/N_2 = 20/80, **1c** (0.5 mol L^{-1}), NBu_4NO_3 (0.5 eq.)	22%	1%	4%
22	O_2/N_2 = 20/80, 100 rpm	8%	4%	0%
23	O_2/N_2 = 20/80, 200 rpm	17%	2%	1%
24	O_2/N_2 = 20/80, 500 rpm	8%	2%	0%
25	O_2/N_2 = 20/80, 600 rpm	7%	1%	0%
26	O_2/N_2 = 20/80, 5 °C	27%	1%	4%
27	O_2/N_2 = 20/80, 50 °C	27%	1%	7%
28	O_2/N_2 = 20/80, NBu_4BF_4 (0.5 eq.)	3%	2%	1%
29	O_2/N_2 = 20/80, NBu_4PF_6 (0.5 eq.)	3%	2%	1%
30	O_2/N_2 = 20/80, NBu_4ClO_4 (0.5 eq.)	4%	3%	2%
31	O_2/N_2 = 20/80, MeCN/H_2O (5 mL, 10 vol.% H_2O)	16%[d]	2%	2%
32	O_2/N_2 = 20/80, MeCN/H_2O (5 mL, 20 vol.% H_2O)	12%[d]	3%	1%
33	O_2/N_2 = 20/80, MeCN/H_2O (5 mL, 10 vol.% H_2O), $NaNO_3$ (0,5 eq.)	15%[d]	0%	3%
34	O_2/N_2 = 20/80, MeCN/H_2O (5 mL, 20 vol.% H_2O), $NaNO_3$ (0,5 eq.)	11%[d]	2%	1%
35	O_2/N_2 = 20/80, isobutyronitrile (5 mL)	24%	1%	3%
36	O_2/N_2 = 20/80, acetone (5 mL)	29%	2%	4%
37	Isobutyronitrile (5 mL)	19%	1%	2%
38	1-Nitropropane (5 mL)	17%[d]	2%	2%
39	O_2/N_2 = 20/80, BDD ‖ BDD	20%	2%	3%
40	O_2/N_2 = 20/80, graphite ‖ graphite	15%	1%	2%
41	Hexadecyltrimethylammonium nitrate (0.5 eq.)	21%[d]	1%	3%
42	1-Butyl-3-methylimidazolium nitrate (0.5 eq.)	17%[d]	0%	8%
43	Methyltrioctylammonium nitrate (0.5 eq.)	28%[d]	0%	4%
44	Tetrabutylphosphonium nitrate (0.5 eq.)	20%[d]	0%	7%

[a.] Undivided 5 mL PTFE cell, glassy carbon electrodes, acetonitrile (5 mL), **1c** (0.2 mol L^{-1}), NBu_4NO_3 (0.5 eq.), 30 °C, O_2 atm. (100 vol.%), 350 rpm, 4 *F*, 10 mA cm^{-2}. [b.] Yield determination via ^{1}H NMR (1,3,5-trimethoxybenzene as internal standard). [c.] Yield determination via GC integrals calculated based on yield of **2c**. [d.] Yield determination via GC (external calibration of **2c**, 1,3,5-trimethoxybenzene as internal standard).

2.3 Optimization for cycloalkene oxidation

Supplementary Table 2: Optimization reactions for cyclododecene (**5c**) oxidation, according to GP 1.

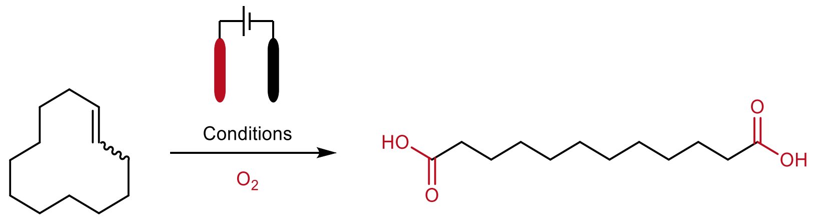

5c, *cis-/trans*-mixture

6c

Entry	Deviation from standard conditions[a]	6c[b]
1	None	68%
2	5 °C, 8 *F*	53%
3	22 °C, **5c** (0.1 mol L^{-1}), 8 *F*	69%
4	22 °C, **5c** (0.1 mol L^{-1}), 6 *F*, 5 mA cm^{-2}	70%
5	22 °C, **5c** (0.1 mol L^{-1}), NBu_4NO_3 (1.0 eq.), 8 *F*	61%
6	22 °C, **5c** (0.1 mol L^{-1}), NBu_4NO_3 (2.0 eq.), 8 *F*	71%
7	22 °C, **5c** (0.1 mol L^{-1}), NBu_4NO_3 (1.0 eq.), 8 *F*, 5 mA cm^{-2}	69%
8	22 °C, **5c** (0.1 mol L^{-1}), NBu_4NO_3 (1.0 eq.), 6 *F*, 5 mA cm^{-2}	66%
9	22 °C, **5c** (0.1 mol L^{-1}), NBu_4NO_3 (1.0 eq.), 4 *F*, 5 mA cm^{-2}	68%
10	22 °C, **5c** (0.075 mol L^{-1}), NBu_4NO_3 (1.3 eq.), 8 *F*	73%
11	22 °C, **5c** (0.05 mol L^{-1}), NBu_4NO_3 (2.0 eq.), 8 *F*	76%
12	22 °C, **5c** (0.05 mol L^{-1})	73%
13	22 °C, **5c** (0.05 mol L^{-1}), 2 *F*	63%
14	22 °C, **5c** (0.05 mol L^{-1}), 5 mA cm^{-2}	75%
15	35 °C, **5c** (0.05 mol L^{-1}), 5 mA cm^{-2}	78%
16	5 °C, **5c** (0.05 mol L^{-1}), 5 mA cm^{-2}	71%
17	50 °C, **5c** (0.05 mol L^{-1}), 5 mA cm^{-2}	57%
18	35 °C, **5c** (0.05 mol L^{-1}), 5 mA cm^{-2}, 200 rpm	63%
19	35 °C, **5c** (0.05 mol L^{-1}), 5 mA cm^{-2}, 500 rpm	59%
20	35 °C, **5c** (0.02 mol L^{-1}), 5 mA cm^{-2}	61%
21	22 °C, **5c** (0.05 mol L^{-1}), no electric current	0%

[a.]Undivided 5 mL PTFE cell, glassy carbon electrodes, isobutyronitrile (5 mL), **5c** (0.2 mol L^{-1}), NBu_4NO_3 (0.5 eq.), 30 °C, O_2 atm. (100 vol.%), 350 rpm, 4 *F*, 10 mA cm^{-2}. [b.]Isolated yields.

2.4 Co-electrolysis reactions for cyclooctane (1c) and cyclooctene (5b)

Supplementary Table 3: Reactions for cyclooctane (**1c**) and cyclooctene (**5b**) co-electrolysis, according to GP 1.

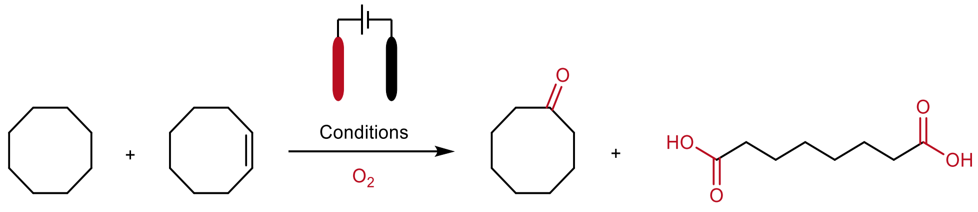

Entry	Mol ratio 1c : 5b	2c[d]	6b[e]
1	1 : 9[a]	4%	40%
2	1 : 3[b]	2%	44%
3	1 : 1[c]	2%	45%

Conditions: Undivided 5 mL PTFE cell, glassy carbon electrodes, acetonitrile (5 mL), NBu_4NO_3 (0.1 mol L^{-1}), 22 °C, O_2 atm. (100 vol.%), 350 rpm, 10 mA cm^{-2}. [a.] **1c** 0.02 mol L^{-1}, **5b** 0.18 mol L^{-1}, 7,6 *F*. [b.] **1c** 0.05 mol L^{-1}, **5b** 0.15 mol L^{-1}, 7 *F*. [c.] **1c** 0.1 mol L^{-1}, **5b** 0.1 mol L^{-1}, 6 *F*. [d.] Yields determined via GC-calibration and refer to mol% of **1c**. [e.] Isolated yields refer to mol% of **5b**.

2.5 Optimization for cycloalkene oxidation in flow

Supplementary Table 4: Optimization reactions for cyclododecene (**5c**) oxidation, according to GP 4.

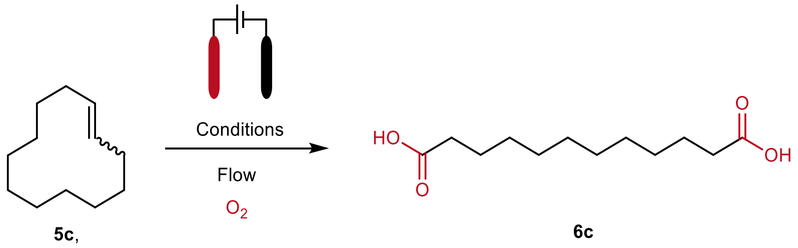

Entry	Deviation from standard conditions[a]	6c[b]
1	None	76%
2	*i*-PrCN (4 mL), **5c** (1.0 mol L^{-1}, 0.96 mL), NBu_4NO_3 (0.25 eq.), 2 *F*, 20 mA cm^{-2}	16%
3	DMC (4 mL), O_2 flow rate: 20 mL min^{-1}, **5c** (1.0 mol L^{-1}, 1 mL), 2 *F*, 20 mA cm^{-2}	10%
4	DMC (4.5 mL), O_2 flow rate: 20 mL min^{-1}, **5c** (0.5 mol L^{-1}, 0.48 mL), 2 *F*, 10 mA cm^{-2}	35%
5	DMC (4.27 mL) + MeOH (0.24 mL), electrolyte flowrate: 5 mL min^{-1}, **5c** (0.5 mol L^{-1}, 0.48 mL), NBu_4NO_3 (0.4 eq.), 2 *F*	33%
6	DMC (4.27 mL) + *i*-PrOH (0.24 mL), O_2 flow rate: 20 mL min^{-1}, electrolyte flowrate: 18 mL min^{-1}, **5c** (0.5 mol L^{-1}, 0.48 mL), NBu_4NO_3 (1.0 eq.), 2 *F*, 20 mA cm^{-2}	38%
7	DMC (8.2 mL) + *i*-PrOH (0.92 mL), 50 °C, O_2 flow rate: 20 mL min^{-1}, electrolyte flowrate: 18 mL min^{-1}, **5c** (0.5 mol L^{-1}, 0.96 mL), NBu_4NO_3 (1.0 eq.), 2 *F*, 20 mA cm^{-2}	52%

[a.] Undivided flow cell, glassy carbon electrodes (2 x 6 cm^2), isobutyronitrile (10 mL), **5c** (0.05 mol L^{-1}), NBu_4NO_3 (0.5 eq.), 20–22 °C, O_2 (100 vol.%) flow rate: 10 mL min^{-1}, electrolyte flowrate: 10 mL min^{-1}, 4 *F*, 5 mA cm^{-2}. [b.] Isolated yields.

2.6 Optimization for benzylic oxidation

Supplementary Table 5: Optimization reactions for the benzylic oxidation, according to GP 1.

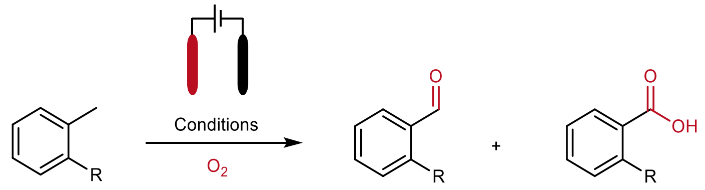

Entry	R	Deviation from standard conditions[a]	13'	14
1	H	None	4%	33%
2	H	5 mA cm^{-2}	15%	3%
3	H	15 mA cm^{-2}	1%	41%
4	H	25 mA cm^{-2}	3%	39%
5	H	30 mA cm^{-2}	3%	37%
6	H	45 mA cm^{-2}	14%	25%
7	H	60 mA cm^{-2}	5%	11%
8	H	5 °C, 15 mA cm^{-2}	1%	35%
9	H	33 °C, **12a** (0.1 mol L^{-1}), NBu_4NO_3 (1.0 eq.), 6 *F*, 30 mA cm^{-2}	6%	42%
10	CH_3	33 °C, **12b** (0.02 mol L^{-1}), NBu_4NO_3 (1.0 eq.), 5 *F*	74%	7%
11	CH_3	33 °C, NBu_4NO_3 (1.0 eq.), 3,2 *F*	27%	0%
12	CH_3	33 °C, NBu_4NO_3 (1.0 eq.), 5 *F*, 20 mA cm^{-2}	45%	1%

[a] Undivided 5 mL PTFE cell, glassy carbon electrodes, acetonitrile (5 mL), **12** (0.2 mol L^{-1}), NBu_4NO_3 (0.5 eq.), 25 °C, O_2 atm. (100 vol.%), 350 rpm, 7 *F*, 10 mA cm^{-2}. Yield determination via ^{1}H NMR (1,3,5-trimethoxybenzene as internal standard).

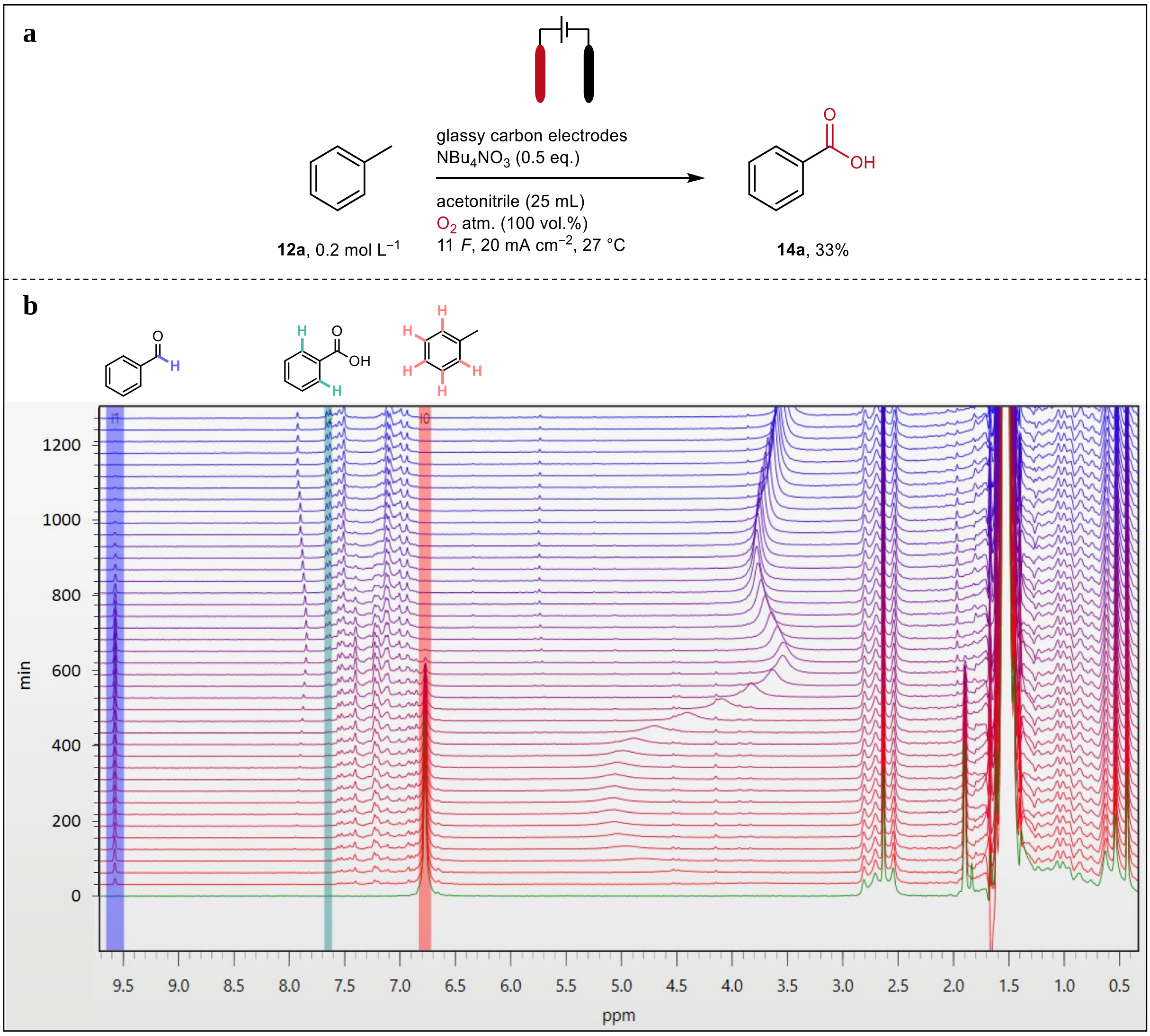

Supplementary Fig. 8: **a** The reaction was performed under the given conditions in a 25 mL beaker-type cell. **b** Stacked ^{1}H NMR spectra for reaction control with a Spinsolv 60 benchtop NMR spectrometer. Signals for integration were chosen in a way that no overlapping occurs. For normalizing the signal intensities to one proton, the toluene (**12a**) proton intensity was divided by 5. The integrated signals for benzaldehyde (**13’a**) and benzoic acid (**14a**) correspond to one proton. Corresponding integration areas are marked as follows: **12a** pink, **13’a** blue, and **14a** green.

2.7 Recovery and reuse of the supporting electrolyte

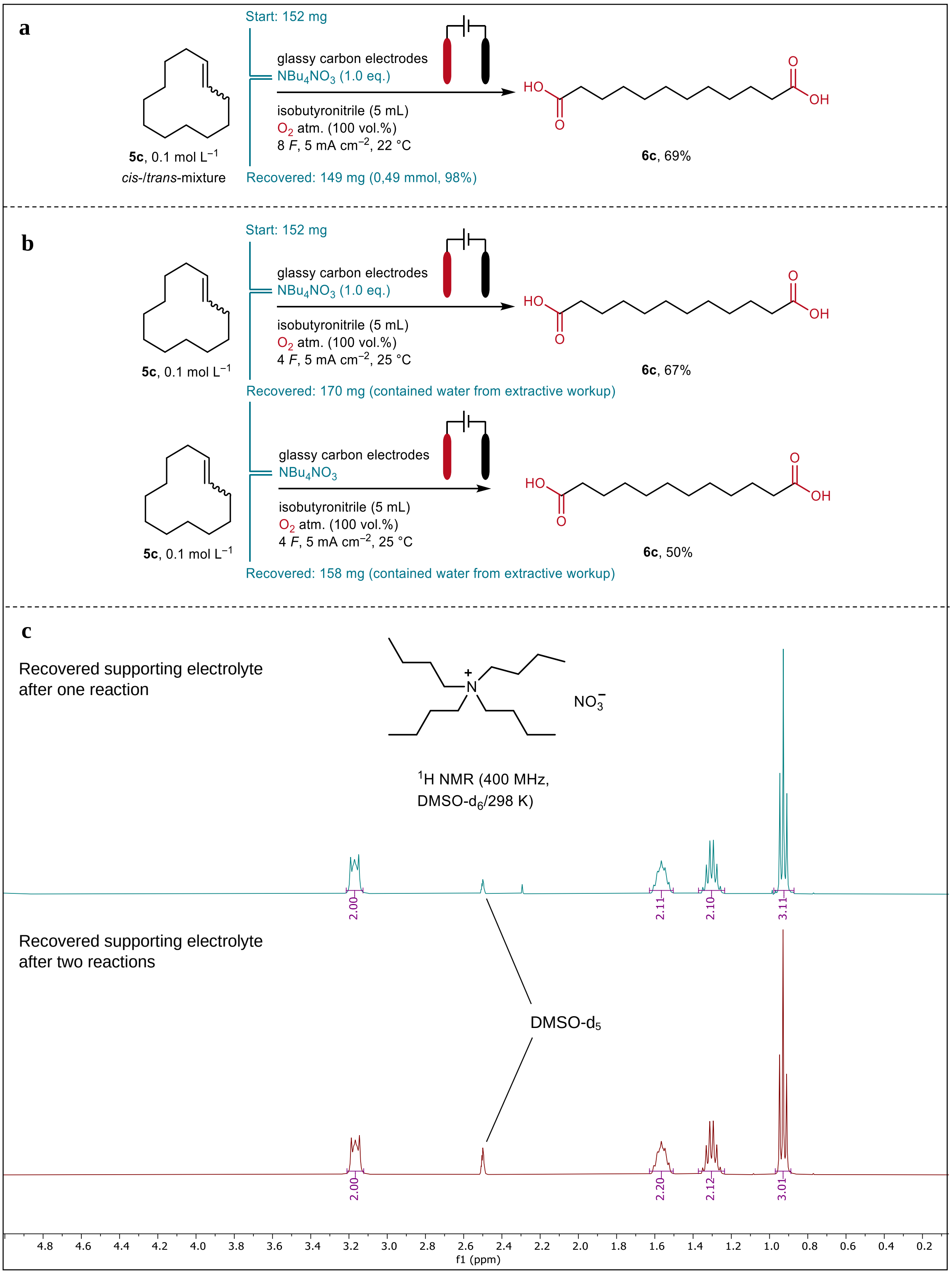

Supplementary Fig. 9: **a** Recovered supporting electrolyte from the aqueous layer of an extractive workup, after one reaction of cyclododecene (**5c**) to dodecanedioic acid (**6c**). **b** Recovery and reuse of supporting electrolyte for a second reaction. **c** Stacked ^{1}H NMR spectra of recovered NBu_4NO_3 supporting electrolyte after one reaction (upper spectrum, blue) and two reactions (lower spectrum, brown).

The supporting electrolyte recovery and recycle was demonstrated in two experiments for synthesizing dodecanedioic acid (**6c**) from cyclododecene (**5c**). During the workup procedure for (di)carboxylic acid and benzoic acid synthesis (see Method section in the manuscript) the supporting electrolyte was recovered from the aqueous layers as a colorless, wax-like solid (149 mg, 0.49 mmol, 98%) (see Supplementary Fig. 9a). The ^{1}H NMR spectrum of the supporting electrolyte is shown in Supplementary Fig. 9c (upper spectrum). In an additional experiment, the supporting electrolyte was recovered after the first electrolysis and reused in a second one to demonstrate recyclability (see Supplementary Fig. 9b). Here, a yield loss of 17% was observed for dodecanedioic acid (**6c**), which could be due to residual water still present in the electrolyte. After reusing the supporting electrolyte, 158 mg was obtained after the second reaction, presumably with residual water after the workup. When comparing the two spectra, it can be seen that no significant organic impurities are present even after the reuse of the supporting electrolyte (see Supplementary Fig. 9c).

2.8 Oxo-functionalization results with branched cycloalkanes

Regioselectivities were investigated on various branched cycloalkanes by GC-FID and GC-MS measurements. The selectivities were determined as GC integral ratios.

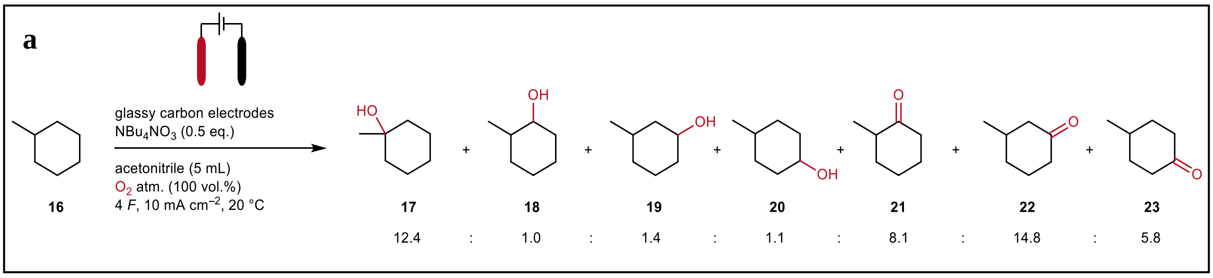

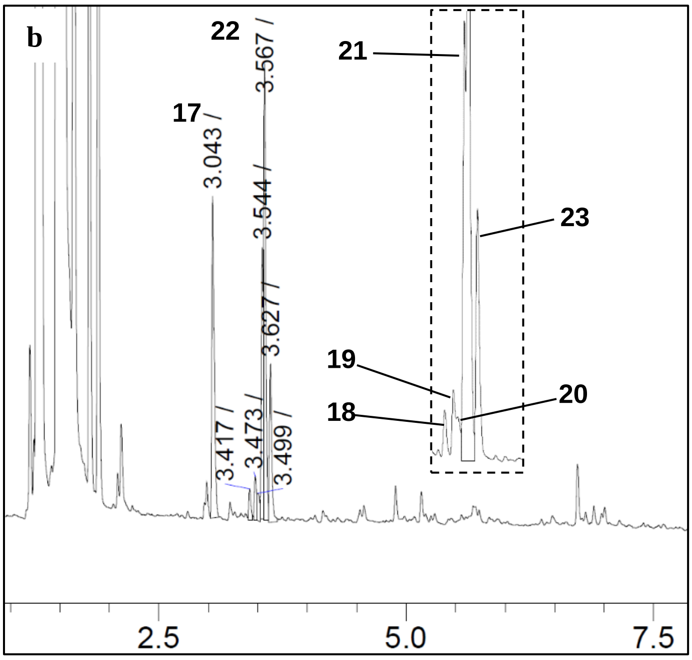

c

Molecule	Ret. time (min)	GC int.	Selectivity ratio
**17**	3.043	8594	12.4
**18**	3.417	693	1.0
**19**	3.473	996	1.4
**20**	3.499	736	1.1
**21**	3.544	5624	8.1
**22**	3.567	10236	14.8
**23**	3.627	3990	5.8

Supplementary Fig. 10: **a** The reaction of methylcyclohexane (**17**) was performed under the given conditions in a 5 mL PTFE cell. **b** GC-FID chromatogram after the electrolysis. The molecules' assignment has been carried out with GC-MS by comparison of the observed mass spectra with the NIST17 mass spectral library entries. **c** Data of the GC-FID chromatogram to evaluate the selectivity ratios for the assigned products.

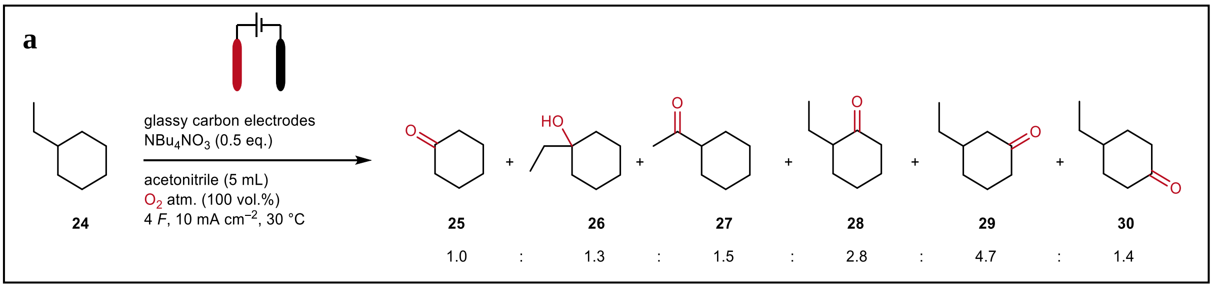

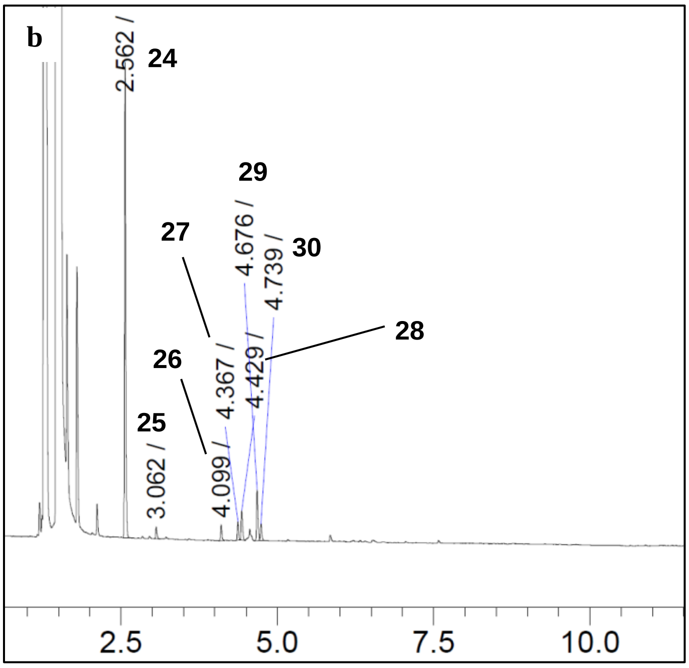

c

Molecule	Ret. time (min)	GC int.	Selectivity ratio
**25**	3.062	1072	1.0
**26**	4.099	1447	1.3
**27**	4.367	1613	1.5
**28**	4.429	2995	2.8
**29**	4.676	5036	4.7
**30**	4.739	1550	1.4

Supplementary Fig. 11: **a** The reaction of ethylcyclohexane (**24**) was performed under the given conditions in a 5 mL PTFE cell. **b** GC-FID chromatogram after the electrolysis. The molecules' assignment has been carried out with GC-MS by comparison of the observed mass spectra with the NIST17 mass spectral library entries. **c** Data of the GC-FID chromatogram to evaluate the selectivity ratios for the assigned products.

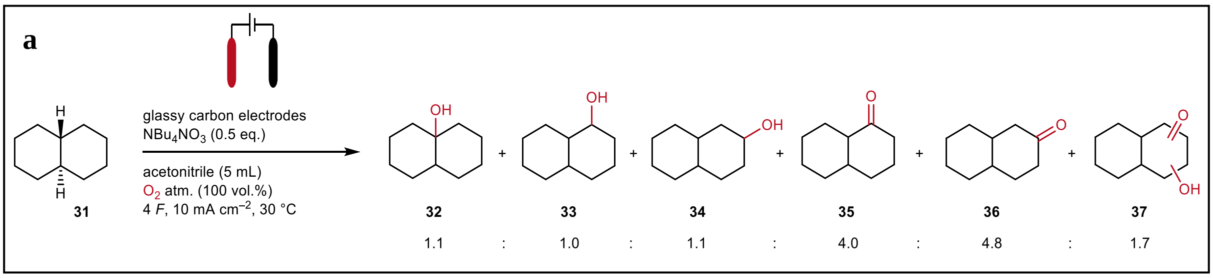

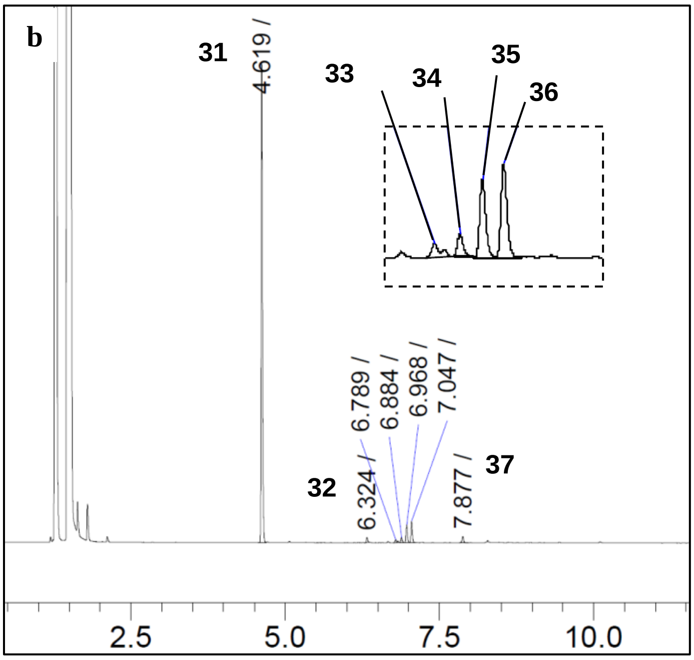

c

Molecule	Ret. time (min)	GC int.	Selectivity ratio
**32**	6.324	3073	1.1
**33**	6.789	2914	1.0
**34**	6.884	3350	1.1
**35**	6.968	11789	4.0
**36**	7.047	14127	4.8
**37**	7.877	4849	1.7

Supplementary Fig. 12: **a** The reaction of *trans*-decalin (**31**) was performed under the given conditions in a 5 mL PTFE cell. **b** GC-FID chromatogram after the electrolysis. The molecules' assignment has been carried out with GC-MS by comparison of the observed mass spectra with the NIST17 mass spectral library entries. **c** Data of the GC-FID chromatogram to evaluate the selectivity ratios for the assigned products.

2.9 Qualitative HRMS analysis of the reaction mixture for diacid synthesis

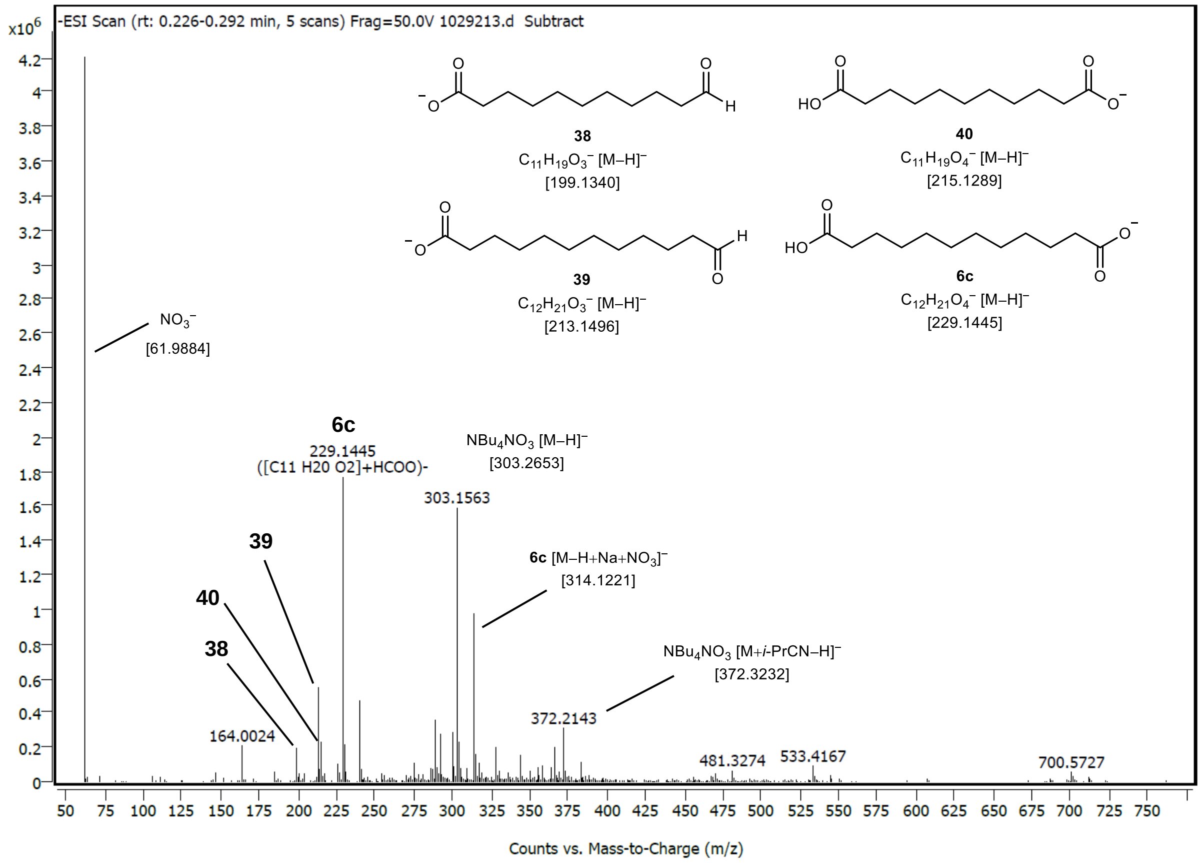

Supplementary Fig. 13: (ESI−)-recorded mass spectra of an HRMS analysis from the reaction solution after electrolysis of cyclododecene (**5c**) to dodecanedioic acid (**6c**) in an isobutyronitrile/NBu_4NO_3 electrolyte.

3. Supplementary Notes

3.1 Determination of dissolved oxygen concentration

Cyclic voltammetry measurements were carried out in a 10 mL snap-cap vial in which the supporting electrolyte (0.1 mol L^{-1}), and optionally the substrate, was dissolved in acetonitrile (5 mL). The composition of the gas space above the electrolyte was adjusted beforehand by using mass flow controllers (5 vol.% O_2 to 100 vol.% O_2 in N_2) and was introduced via a cannula during the entire measurement time. The temperature control of 25 °C was carried out via an oil bath. Between the individual measurements, the electrolyte was stirred at 400 rpm. Before each measurement, the working electrode was polished with BASi® Electrode Polishing Alumina Suspension (Bioanalytical Systems Inc., West Lafayette, Indiana) for approx. 30 seconds.

The statistical software Origin 7.5 SR6 (OriginLab Corporation, Northampton, Massachusetts) was used for linear regression analysis. Mathematical calculations were performed using Microsoft® Excel® 2019. Voltammograms were recorded using NOVA 2.1.3 software (Metrohm AG, Herisau, Switzerland).

To determine the concentration of dissolved oxygen in acetonitrile at different O_2 vol.% values in the atmosphere, a potentiometric method via cyclic voltammetry was used. Given a known diffusion coefficient D of the investigated species, the Randles-Ševčík equation (1) can be used to determine its concentration. The measured peak current I_p is proportional to the concentration c of the species. Using the relationship, $I_p \sim \sqrt{v}$ with v as scan rate, the concentration can be determined from the slope m of a linear fit via the equations (2) and (3). The propagation of uncertainty has been calculated via equations (4) and (5) which derive from the standard error propagation equation[3].

Randles-Ševčík equation for 25 °C:

$$j_p = \frac{I_p}{A} = 2.69 \cdot 10^5 c\sqrt{n^3 D v} \qquad (1)$$

$$m = 2.69 \cdot 10^5 c\sqrt{n^3 D} \qquad (2)$$

$$c = \frac{m}{2.69 \cdot 10^5 \sqrt{n^3 D}} \qquad (3)$$

j_p: Maximum of current density [A cm^{-2}]
I_p: Current maximum [A]
A: Electrode area [cm^2]
c: Concentration [mol mL^{-1}]
D: Diffusion coefficient [cm^2 s^{-1}]
v: Scan rate [V s^{-1}]
n: Number of transferred electrons ($n = 1$)
m: Slope of the linear fit of $j_p(\sqrt{v})$

$$\Delta j_p = \Delta \frac{I_p}{A} = \frac{\partial j_p}{\partial I_p}\Delta I_p + \frac{\partial j_p}{\partial A}\Delta A = \frac{\Delta I_p}{A} - \frac{I_p}{A^2}\Delta A \qquad (4)$$

$$\Delta c = \frac{\partial c}{\partial m}\Delta m + \frac{\partial c}{\partial D}\Delta D = \frac{\Delta m}{2.69 \cdot 10^5 \sqrt{n^3 D}} - \frac{m \Delta D}{2 \cdot 2.69 \cdot 10^5 \sqrt{n^3 D^3}} \qquad (5)$$

As diffusion coefficient of dissolved oxygen, a literature known value of $2.1 \cdot 10^{-5}$ cm^2 s^{-1} (in acetonitrile/NBu_4PF_6 (0.1 mol L^{-1})) was used[4]. Cyclic voltammetry has been performed using the conditions in Supplementary Fig. 10d. The values for I_p in Supplementary Table 6 are mean values out of three measurements, each with an associated uncertainty ΔI_p. j_p and Δj_p were calculated using equations (1) and (4). c and Δc were calculated regarding equations (3) and (5). Supplementary Fig. 10 shows furthermore the corresponding plots of j_p vs. $v^{1/2}$ including the linear regressions as well as the dependence of the dissolved oxygen concentration $c(O_2)$ on the atmospheric O_2 content.

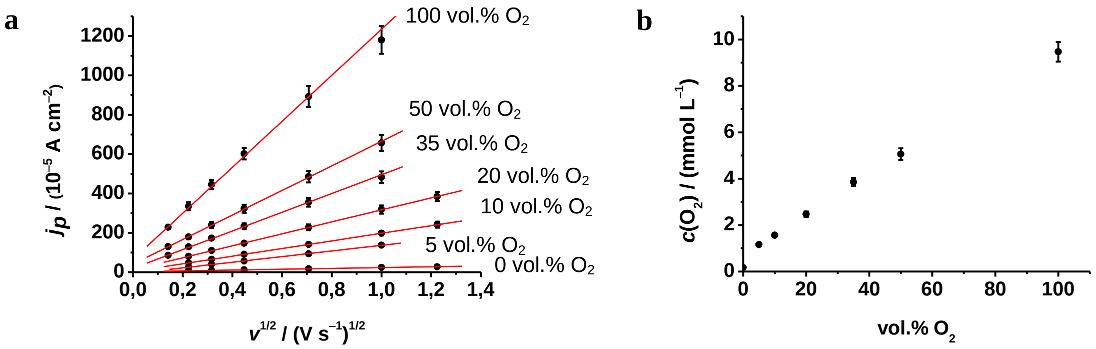

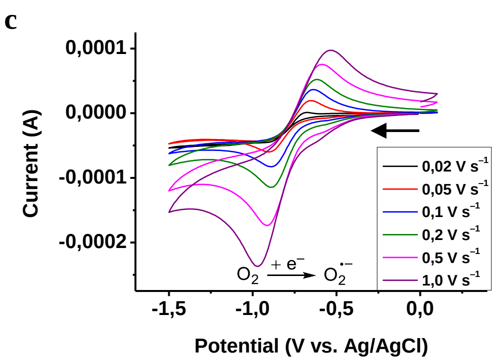

d

Condition	Value/Specification
Temperature	25 °C
Atmosphere	Air / 0-100 vol.% O_2
Supp. electr.	NBu_4NO_3 (0.1 mol L^{-1})
Solvent	Acetonitrile (5 mL)
$D(O_2)$	$2.1 \cdot 10^{-5}$ $cm^2\,s^{-1}$
	(Uncertainty not given, $\Delta D(O_2) = 0$ $cm^2\,s^{-1}$)
n	1
WE	Glassy carbon disk
CE	Glassy carbon rod
RE	Ag/AgCl in ethanol sat. with LiCl
Area WE	0.071 ± 0.005 cm^2
Voltage range	-1.5 V to 0.1 V

Supplementary Fig. 14: **a** Plot j_p vs. $v^{1/2}$ for the O_2 measurements in MeCN/NBu_4NO_3 at different atmospheric O_2 contents. Error bars along the *y*-axis are calculated via uncertainty propagation. Linear regression straight lines are labeled in red. **b** Dependence of dissolved oxygen concentration $c(O_2)$ in MeCN on the atmospheric O_2 content. Error bars along the *y*-axis are calculated via uncertainty propagation. **c** Exemplarily cyclic voltammograms for oxygen reduction at different scan rates at air atmosphere. CVs at the remaining atmosphere constitutions were measured and recorded analogously. **d** Cyclic voltammetry conditions for dissolved oxygen concentration determination.

The resulting values for the dissolved oxygen concentrations (at air: (2.4 ± 0.1) mmol L^{-1}; at 100 vol.% O_2: (9.5 ± 0.6) mmol L^{-1}) are comparable to the ones, which are literature described (at air: (2.42 ± 0.14) mmol L^{-1} (photochemical determination)[5], at 100 vol.% O_2: (8.1 ± 0.6) mmol L^{-1} (determination via GC)[6]). In contrast to the latter one, for the determination in this work, O_2 was permanently introduced into the atmosphere above the electrolyte during the measurements.

With regards to the Supplementary Table 1 (entry 10) a minimum dissolved oxygen concentration in the electrolyte of approx. 1 mmol L^{-1} (5 vol.% O_2 in the atmosphere) is necessary for the cycloalkane oxidation to occur.

Supplementary Table 6: Current maximums at different scan rates for O_2 in MeCN/NBu_4NO_3 including the calculated O_2 concentrations.

Atm. (vol.% O_2)	I_p / (10^{-5} A)	v / (V s^{-1})	$\sqrt{v}$ / $\sqrt{\text{V s}^{-1}}$	j_p / (10^{-5} A cm^{-2})	m / $\left(10^{-5}\ \frac{\text{mol}}{\text{mL}}\sqrt{\frac{\text{cm}^2}{\text{s}}}\right)$	**$c(O_2)$ / (mmol L^{-1})**
Air	4.4 ± 0.2	0.02	0.14	62 ± 2	297 ± 14	**2.4 ± 0.1**
	5.57 ± 0.01	0.05	0.22	79 ± 5		
	7.62 ± 0.09	0.10	0.32	108 ± 6		
	10.72 ± 0.07	0.20	0.45	152 ± 9		
	16.9 ± 0.1	0.50	0.71	239 ± 14		
	23.2 ± 0.1	1.00	1.00	328 ± 20		
0	0.50 ± 0.02	0.05	0.22	7.1 ± 0.2	21.0 ± 0.5	**0.171 ± 0.004**
	0.62 ± 0.02	0.10	0.32	8.8 ± 0.4		
	0.87 ± 0.04	0.20	0.45	12.3 ± 0.3		
	1.21 ± 0.07	0.50	0.71	17.2 ± 0.2		
	1.74 ± 0.05	1.00	1.00	24.6 ± 0.9		
	1.95 ± 0.06	1.50	1.22	27.5 ± 1.0		
5	1.8 ± 0.2	0.05	0.22	25.5 ± 0.5	143 ± 6	**1.16 ± 0.05**
	2.84 ± 0.01	0.10	0.32	40 ± 3		
	4.02 ± 0.02	0.20	0.45	57 ± 3		
	6.60 ± 0.02	0.50	0.71	93 ± 6		
	9.7 ± 0.2	1.00	1.00	137 ± 7		
10	3.4 ± 0.1	0.05	0.22	48 ± 2	194 ± 9	**1.57 ± 0.07**
	4.59 ± 0.02	0.10	0.32	65 ± 4		
	6.42 ± 0.04	0.20	0.45	91 ± 5		
	10.0 ± 0.1	0.50	0.71	141 ± 8		
	14.0 ± 0.1	1.00	1.00	198 ± 11		
	17.09 ± 0.03	1.50	1.22	242 ± 16		
20	5.7 ± 0.2	0.05	0.22	81 ± 3	304 ± 15	**2.5 ± 0.1**
	7.76 ± 0.04	0.10	0.32	110 ± 7		
	10.41 ± 0.02	0.20	0.45	147 ± 9		
	16.11 ± 0.04	0.50	0.71	228 ± 15		
	22.51 ± 0.03	1.00	1.00	318 ± 21		
	27.1 ± 0.2	1.50	1.22	383 ± 23		
35	6.1 ± 0.2	0.02	0.14	86 ± 3	475 ± 22	**3.9 ± 0.2**
	9.12 ± 0.06	0.05	0.22	129 ± 8		
	12.2 ± 0.1	0.10	0.32	173 ± 10		
	16.50 ± 0.07	0.20	0.45	233 ± 15		
	25.1 ± 0.1	0.50	0.71	355 ± 22		
	34.1 ± 0.2	1.00	1.00	483 ± 30		
50	9.2 ± 0.3	0.02	0.14	130 ± 4	624 ± 31	**5.1 ± 0.3**
	12.67 ± 0.07	0.05	0.22	179 ± 11		
	16.96 ± 0.04	0.10	0.32	240 ± 15		
	22.80 ± 0.06	0.20	0.45	323 ± 21		
	34.3 ± 0.2	0.50	0.71	486 ± 29		
	46.5 ± 0.2	1.00	1.00	657 ± 41		
100	16.2 ± 0.5	0.02	0.14	229 ± 8	1168 ± 52	**9.5 ± 0.4**
	23.7 ± 0.1	0.05	0.22	335 ± 21		
	31.5 ± 0.4	0.10	0.32	445 ± 24		
	42.5 ± 0.8	0.20	0.45	602 ± 29		
	63.1 ± 0.4	0.50	0.71	892 ± 53		
	83.4 ± 0.6	1.00	1.00	1180 ± 71		

3.2 Mechanism elucidation experiments

3.2.1 Griess test

Griess test for nitrite detection was conducted according to the following procedure:

Solution A and B were freshly prepared before the test. Solution A: in a 10 mL round bottom flask sulfanilic acid (20 mg) is dissolved in aqueous acetic acid (30%, 2,5 mL). Solution B: In a 10 mL round bottom flask 1-naphthylamine (25 mg) is dissolved in aqueous acetic acid (30%, 2,5 mL). Both solutions can be stored at 4 °C. Prior to the test, 1–2 drops out of each solution are combined and mixed in a reagent tube to a colorless liquid. One drop of the sample is added to the reagent tube.

Result: If the solution color turns to red/pink, the test is positive for nitrite, which is present in the sample. Coloring occurs due to the formation of an azo dye compound. Nitrate instead does not lead to a positive result and the reaction solution stays colorless.

3.2.2 Ion chromatography

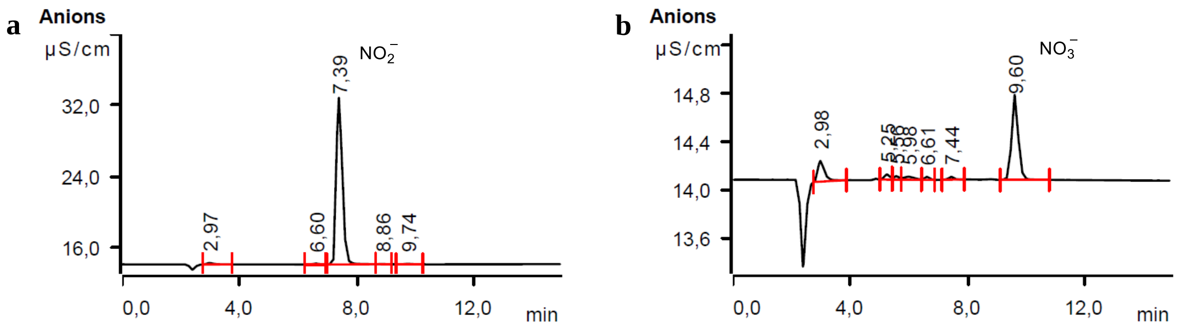

Supplementary Fig. 15: **a** Anion chromatogram of sodium nitrite (0.01 mg mL^{-1} in deionized water) as reference. **b** Anion chromatogram of the aqueous layer after extractive work-up from the reaction solution.

3.2.3 pH test

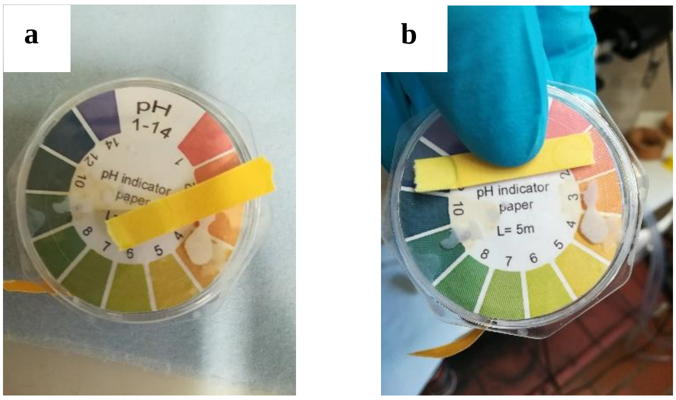

Supplementary Fig. 16: **a** After electrolysis under 100 vol.% oxygen atmosphere: pH 5–6. **b** After electrolysis under 100 vol.% argon atmosphere: pH 8–9.

3.2.4 Peroxide test with titanyl sulfate

Peroxide test for H_2O_2 and organic peroxide detection was conducted after the following procedure:

In a reagent tube titanyl sulfate (10 mg) is suspended in 5 drops of concentrated sulfuric acid. Afterwards, 1 mL of the analysis solution is added into the reagent tube.

Result: if peroxide species are present in the analysis solution the color turns from colorless to yellow/orange, indicating the formation of peroxotitanyl ion $(TiO_2)^{2+}$.

Electrolysis was performed regarding GP 1: the reaction was stopped manually at 50 C and the peroxide test was performed immediately afterwards. Conditions: acetonitrile (5 mL), NBu_4NO_3 (0.1 mol L^{-1}), 10 mA cm^{-2}, under 100 vol.% O_2, once with substrate (cyclooctane (**1c**), 0.2 mol L^{-1}) and once without substrate.

3.2.5 Karl Fischer titration

For the determination of an increased water content after electrolysis, a coulometric Karl Fischer titration was performed after the following procedure:

The titration cell was conditioned until the drift was <10 µg min^{-1}. Prior to the analysis samples 1 mL of a water standard 0.1 solution was measured three times resulting to (0.106 ± 0.002) mg g^{-1} (target: (0.100 ± 0.009) mg g^{-1}). The measurements were conducted following the instruction manual. Approx. 0.5 mL of the analysis sample was drawn into a cannula syringe and tared on a fine balance. Immediately after starting the measurement, the sample was injected within 10 seconds into the titration cell without contacting something of the inner parts. The emptied syringe was weighed and the determined weight entered into the software. The procedure was repeated three times per sample.

Supplementary Table 7: Karl Fischer titration after electrolysis, once with cyclooctane (**1c**) substrate, once without.

Sample	Measurement	*m*(H_2O) / weight of sample taken	ppm H_2O	mg g^{-1} H_2O	
Electrolysis with substrate	1	2222.5 µg / 0.496 g	4513.8	4.4808	4.474 ± 0.006
	2	2147.4 µg / 0.480 g	4514.5	4.4738	
	3	2189.6 µg / 0.490 g	4512.4	4.4686	
Electrolysis without substrate	1	2136.2 µg / 0.495 g	4357.7	4.3156	4.315 ± 0.008
	2	2083.6 µg / 0.482 g	4370.3	4.3228	
	3	2071.7 µg / 0.481 g	4353.3	4.3071	

Electrolysis was performed regarding GP 1: After electrolysis the cell content was transferred into a snap cap vial, weighed and sealed with Parafilm® M. Titration of the samples was performed immediately afterwards. Conditions: acetonitrile (5 mL), NBu_4NO_3 (0.1 mol L^{-1}), 10 mA cm^{-2}, 8 *F*, under 100 vol.% O_2, once with substrate (cyclooctane (**1c**), 0.2 mol L^{-1}) and once without substrate.

3.2.6 Control experiment for nitrate radical observation

The reaction was conducted according to GP 1. As anode boron-doped diamond (BDD) and as cathode nickel were used, since both are stable in an acidic environment and nickel has a relatively low overpotential regarding reductive hydrogen evolution reaction[7]. Instead of oxygen, an argon atmosphere was set within the cell to suppress an oxygen reduction reaction. Cylooctene (**5b**, 1 mmol) was subjected to 2 *F* and 10 mA cm^{-2}. After electrolysis, three drops were eluted with ethyl acetate through approx. 330 mg silica 60M and filled into a vial for GC-MS analysis.

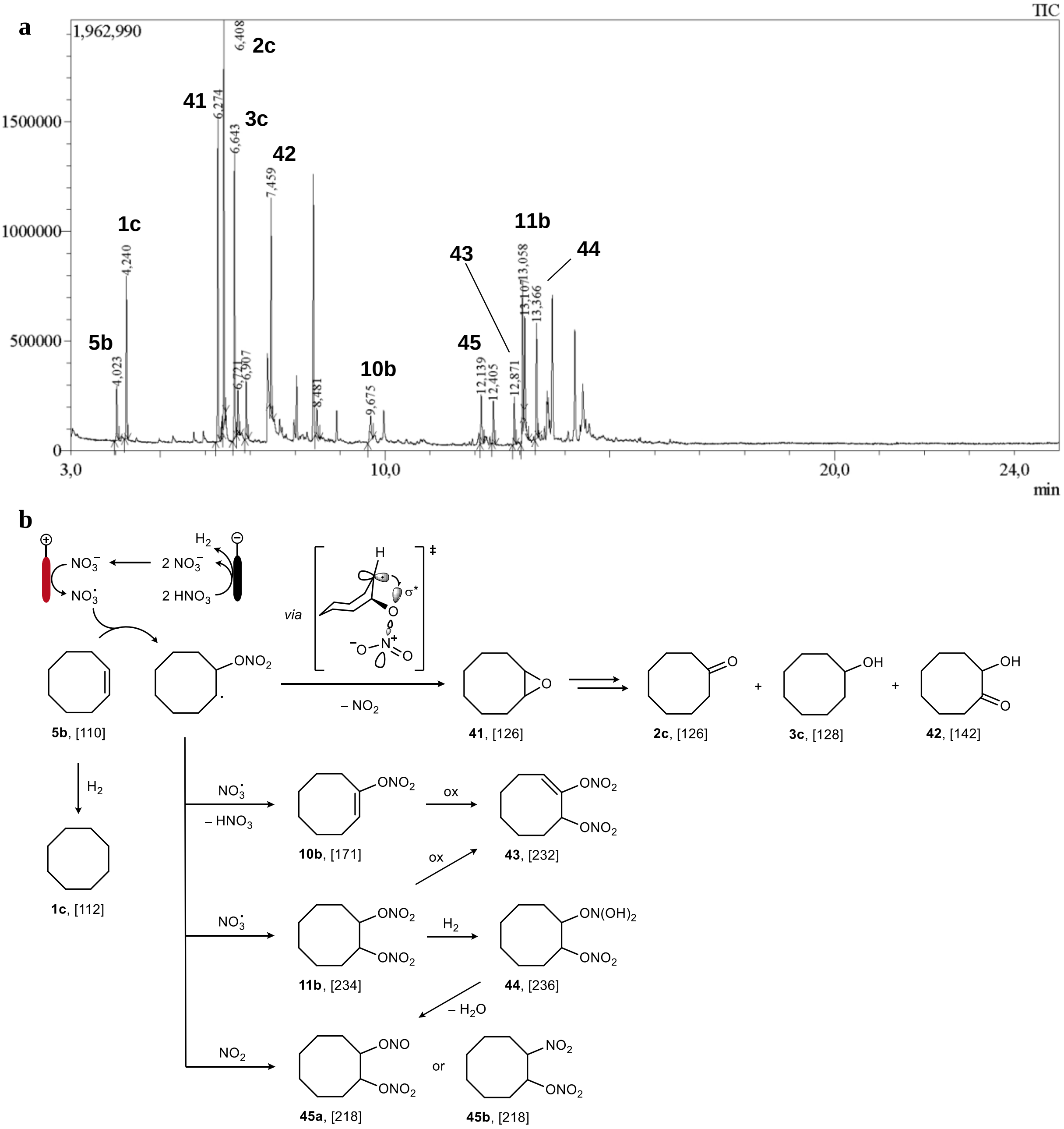

Supplementary Fig. 17: **a** GC-MS of the reaction solution. The molecules' assignment has been carried out by comparison of the observed mass spectra with the NIST17 mass spectral library entries. **b** Mechanistic considerations regarding the observed signals in the GC-MS analysis.

As one of the main products an epoxide **41** was detected. The observation of its formation through the influence of nitrate radicals under argon atmosphere is in accordance to literature reports[8]. The presence of water and the acidic environment due to the use of nitric acid can lead to further oxidized species like **2c**, **3c** and **42**. Nitrate radicals can possibly lead to **10b** via H-abstraction, or **11b** via recombination.

Due to the formation of epoxides, NO_2 radicals are probably present in the reaction solution as well, which could possibly explain the formation of **45** species.

3.2.7 Cyclic voltammetry studies

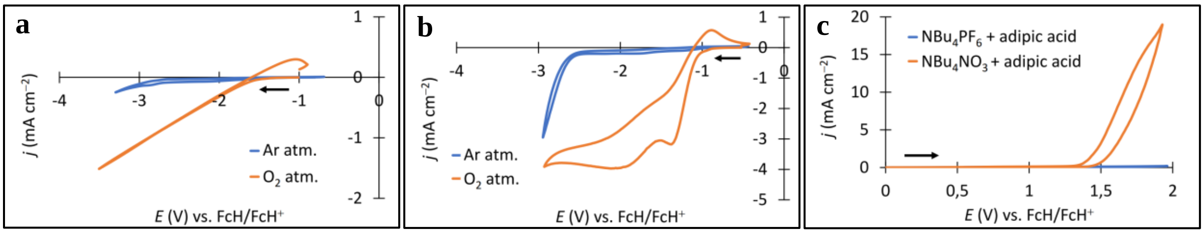

Supplementary Fig. 18: **a** Comparison of the reduction behavior of a dimethyl carbonate/*i*-PrOH (9:1) electrolyte in the presence and absence of oxygen. Electrolyte: dimethyl carbonate (4.5 mL), 2-propanol (0.5 mL), NBu_4NO_3 (0.1 mol L^{-1}). Conditions: glassy carbon disk (working electrode, 3 mm diameter), glassy carbon rod (counter electrode), Ag/AgCl in saturated LiCl/EtOH (reference electrode), Ferrocene/Ferrocenium (FcH/FcH^+) as internal reference ($E_{1/2}$ = 0.79–0.99 V), 50 mV s^{-1}. **b** Comparison of the reduction behavior of an acetonitrile/water (2 vol.%) electrolyte in the presence and absence of oxygen. Electrolyte: acetonitrile (4.9 mL), water (0.9 mL), NBu_4NO_3 (0.1 mol L^{-1}). Conditions: see Supplementary Fig. 18a, ($E_{1/2}$(FcH/FcH^+) = 0.52–0.54 V). **c** Investigation of an adipic acid oxidation in a NO_3^- and a PF_6^- supported electrolyte system. Electrolyte: acetonitrile (5 mL), NBu_4NO_3 or NBu_4PF_6 (0.1 mol L^{-1}), adipic acid (**6a**, 0.01 mol L^{-1}). Conditions: see Supplementary Fig. 18a, measurements were performed under air atmosphere, ($E_{1/2}$(FcH/FcH^+) = 0.54–0.58 V).

3.3 *N*-Acetylbenzamide formation

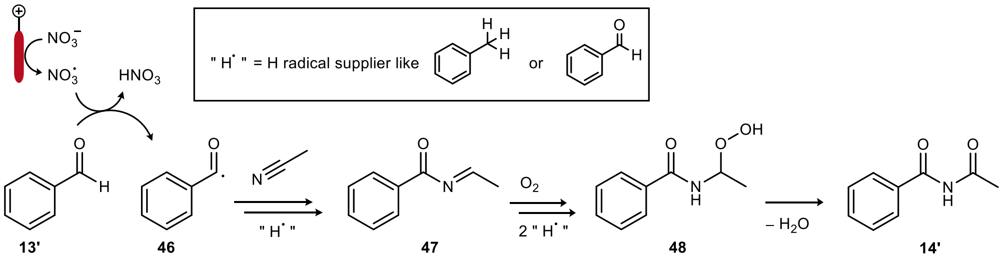

Supplementary Fig. 19: Presumed pathway for *N*-acetylbenzamide formation.

3.4 Syntheses of supporting electrolytes

<u>General procedure:</u> In a 50 mL round-bottom flask silver nitrate (1.1–1.2 eq.) is dissolved in 5 mL deionized water. Alkylammonium (or alkylphosphonium) bromide (1.0 eq.) is dissolved in a water-acetone-mixture and added dropwise to the silver nitrate solution while vigorous stirring. After approx. 10 min, the precipitated silver bromide is filtered and washed with water and acetone. The filtrate is extracted with ethyl acetate, while approx. 300 mg sodium nitrate are added to the aqueous layer for improved phase separation. The organic layer is separated, the solvent is distilled and the remaining product dried under reduced pressure.

Hexadecyltrimethylammonium nitrate

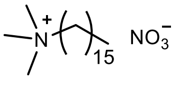

Hexadecyltrimethylammonium bromide (525 mg, 1.44 mmol, 1.0 eq.) is dissolved in 12.5 mL water and 5 mL acetone and is converted according to the general procedure with silver nitrate (294 mg, 1.73 mmol, 1.2 eq.). After workup, the product is obtained as a colorless powder (yield: 53%, 263 mg, 0.76 mmol).

^{1}H NMR (400 MHz, D_2O) δ [ppm] = 3.35–3.31 (m, 2H), 3.13 (s, 9H), 1.80–1.72 (m, 2H), 1.37–1.31 (m, 26H), 0.90–0.87 (m, 3H); ^{13}C NMR (101 MHz, D_2O) δ [ppm] = 66.4, 52.7, 32.1, 30.3, 30.3, 30.3, 30.2, 30.2, 30.1, 30.0, 29.8, 28.7, 29.3, 26.2, 22.8, 22.7, 13.8.

1-Butyl-3-methylimidazolium nitrate

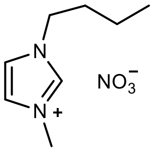

1-Butyl-3-methylimidazolium bromide (543 mg, 2.48 mmol, 1.0 eq.) is dissolved in 2 mL water and 5 mL acetone and is converted according to the general procedure with silver nitrate (463 mg, 2.73 mmol, 1.1 eq.). Due to the product's hydrophily, the aqueous filtrate is distilled directly and the remaining product is dried under reduced pressure. A colorless, highly viscous liquid is obtained (yield: 90%, 446 mg, 2.22 mmol).

^{1}H NMR (400 MHz, D_2O) δ [ppm] = 8.73 (s, 1H), 7.50 (dd, J = 2.2 Hz, J = 1.8 Hz, 1H), 7.45 (dd, J = 2.2 Hz, J = 1.8 Hz, 1H), 4.21 (t, J = 7.3 Hz, 2H), 3.92 (s, 3H), 1.86 (quint, J = 7.4 Hz, 2H), 1.33 (sextet, J = 7.4 Hz, 2H), 0.93 (t, J = 7.4 Hz, 3H); ^{13}C NMR (101 MHz, D_2O) δ [ppm] = 135.8, 123.4, 122.2, 49.2, 35.5, 31.3, 18.7, 12.6.

Methyltrioctylammonium nitrate

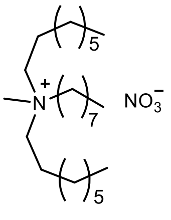

Methyltrioctylammonium bromide (520 mg, 1.16 mmol, 1.0 eq.) is dissolved in 1 mL water and 5 mL acetone and is converted according to the general procedure with silver nitrate (216 mg, 1.28 mmol, 1.1 eq.). After workup, the product is obtained as a highly viscous liquid (yield: 95%, 473 mg, 1.10 mmol).

^{1}H NMR (400 MHz, DMSO-d_6) δ [ppm] = 3.21–3.16 (m, 6H), 2.93 (s, 3H), 1.64–1.56 (m, 6H), 1.32–1.21 (m, 30H), 0.88–0.85 (m, 9H); ^{13}C NMR (101 MHz, DMSO-d_6) δ [ppm] = 60.5, 47.5, 31.2, 28.5, 28.4, 25.8, 22.1, 21.3, 14.0.

Tetrabutylphosphonium nitrate

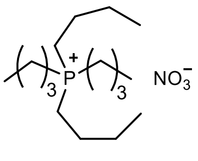

Tetrabutylphosphonium bromide (529 mg, 1.56 mmol, 1.0 eq.) is dissolved in 1 mL water and 5 mL acetone and is converted according to the general procedure with silver nitrate (220 mg, 1.72 mmol, 1.1 eq.). Due to the product's hydrophily, the aqueous filtrate is distilled directly and the remaining product is dried under reduced pressure. A colorless, highly viscous liquid is obtained (yield: 99%, 497 mg, 1.54 mmol).

^{1}H NMR (400 MHz, D_2O) δ [ppm] = 2.19–2.12 (m, 8H), 1.60–1.41 (m, 16H), 0.93 (t, J = 7.2 Hz, 12H); ^{13}C NMR (101 MHz, D_2O) δ [ppm] = 23.3 (d, J = 15.3 Hz), 22.7 (d, J = 4.6 Hz), 17.6 (d, J = 48.3 Hz), 12.5; ^{31}P NMR (162 MHz, D_2O) δ [ppm] = 33.20.

3.5 Characterization of oxo-functionalization products

Cyclohexanone (2a)

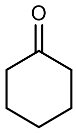

According to the general protocol (GP 2) cyclohexane (0.421 g, 5.00 mmol, 1.0 eq.) and tetrabutylammonium nitrate (0.76 g, 2.50 mmol, 0.5 eq.) are dissolved in acetonitrile (25 mL) and electrolyzed with constant current (10 mA cm^{-2}) at 25 °C, under 100 vol.% oxygen atmosphere and 4 *F*. After with a diethyl ether/water extraction, the product is obtained as colorless liquid (yield: 6%, 30 mg, 0.31 mmol).

R_f (cyclohexane/ethyl acetate = 9:1): 0.40; ^{1}H NMR (400 MHz, $CDCl_3$) δ [ppm] = 2.32–2.29 (m, 4H), 1.86–1.80 (m, 4H), 1.72–1.67 (m, 2H); ^{13}C NMR (101 MHz, $CDCl_3$) δ [ppm] = 212.6, 42.0, 27.1, 25.0; GC-MS (EI): m/z: 98 [M^+], 55 [base peak].

All spectroscopical data match to the reported data[9].

Cycloheptanone (2b)

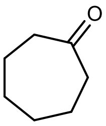

According to the general protocol (GP 2) cycloheptane (0.491 g, 5.00 mmol, 1.0 eq.) and tetrabutylammonium nitrate (0.76 g, 2.50 mmol, 0.5 eq.) are dissolved in acetonitrile (25 mL) and electrolyzed with constant current (10 mA cm^{-2}) at 27 °C, under 100 vol.% oxygen atmosphere and 4 *F*. After work-up with a diethyl ether/water extraction, the product is obtained as a colorless liquid (yield: 16%, 0.090 g, 0.80 mmol).

R_f (cyclohexane/ethyl acetate = 9:1): 0.40; ^{1}H NMR (400 MHz, $CDCl_3$) δ [ppm] = 2.50–2.47 (m, 4H), 1.73–1.64 (m, 8H); ^{13}C NMR (101 MHz, $CDCl_3$) δ [ppm] = 215.9, 44.0, 30.6, 24.5; GC-MS (EI): m/z: 112 [M^+], 55 [base peak].

All spectroscopical data match to the reported data[10].

Cyclooctanone (2c)

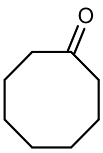

According to the general protocol (GP 2) cyclooctane (0.561 g, 5.00 mmol, 1.0 eq.) and tetrabutylammonium nitrate (0.76 g, 2.50 mmol, 0.5 eq.) are dissolved in acetonitrile (25 mL) and electrolyzed with constant current (10 mA cm^{-2}) at 30 °C, under 100 vol.% oxygen atmosphere and 4 *F*. After work-up with a cyclohexane/water extraction, the product is obtained as colorless liquid (yield: 42%, 0.261 g, 2.07 mmol).

R_f (cyclohexane/ethyl acetate = 7:3): 0.66; ^{1}H NMR (400 MHz, $CDCl_3$) δ [ppm] = 2.39–2.36 (m, 4H), 1.87–1.81 (m, 4H), 1.54–1.48 (m, 4H), 1.36–1.31 (m, 2H); ^{13}C NMR (101 MHz, $CDCl_3$) δ [ppm] = 218.5, 42.0, 27.2, 25.7, 24.8; GC-MS (EI): m/z: 126 [M^+], 55 [base peak].

All spectroscopical data match to the reported data[10].

Cyclodecanone (2d)

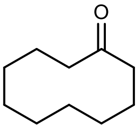

According to the general protocol (GP 2) cyclodecane (0.701 g, 5.00 mmol, 1.0 eq.) and tetrabutylammonium nitrate (0.76 g, 2.50 mmol, 0.5 eq.) are dissolved in acetonitrile (25 mL) and electrolyzed with constant current (10 mA cm^{-2}) at 30 °C, under 100 vol.% oxygen atmosphere and 5 *F*. After removing the solvent in vacuo, the residue was purified by column chromatography (cyclohexane/ethyl acetate = 9:1) to yield the product as a colorless liquid (yield: 12%, 90 mg, 0.59 mmol).

R_f (cyclohexane/ethyl acetate = 9:1): 0.57; ^{1}H NMR (400 MHz, $CDCl_3$) δ [ppm] = 2.51–2.48 (m, 4H), 1.86–1.80 (m, 4H), 1.50–1.44 (m, 4H), 1.36–1.31 (m, 6H); ^{13}C NMR (101 MHz, $CDCl_3$) δ [ppm] = 215.1, 42.1, 25.2, 25.0, 24.9, 23.5; GC-MS (EI): m/z: 154 [M^+], 55 [base peak].

Cyclododecanone (2e)

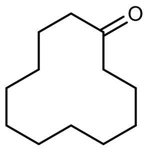

According to the general protocol (GP 2) cyclododecane (0.842 g, 5.00 mmol, 1.0 eq.) and tetrabutylammonium nitrate (0.76 g, 2.50 mmol, 0.5 eq.) are dissolved in isobutyronitrile (25 mL) and electrolyzed with constant current (10 mA cm^{-2}) at 27 °C, under 100 vol.% oxygen atmosphere and 4 *F*. After removing the solvent in vacuo, the residue was purified by column chromatography (cyclohexane/ethyl acetate = 10:0 → 9:1) to yield the product as a colorless solid (yield: 21%, 0.194 g, 1.06 mmol).

R_f (cyclohexane/ethyl acetate = 9:1): 0.48; ^{1}H NMR (400 MHz, $CDCl_3$) δ [ppm] = 2.47–2.44 (m, 4H), 1.74–1.68 (m, 4H), 1.33–1.24 (m, 14H); ^{13}C NMR (101 MHz, $CDCl_3$) δ [ppm] = 213.1, 40.5, 24.9, 24.7, 24.4, 22.7, 22.5; GC-MS (EI): m/z: 182 [M^+], 41 [base peak].

All spectroscopical data match to the reported data[10].

Hexane-1,6-dioic acid (6a)

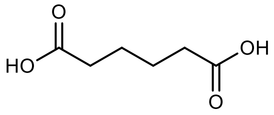

According to the general protocol (GP 1) cyclohexene (21 mg, 0.25 mmol, 1.0 eq.) and tetrabutylammonium nitrate (38 mg, 0.125 mmol, 0.5 eq.) are dissolved in acetonitrile (5 mL) and electrolyzed with constant current (5 mA cm^{-2}) at 35 °C, under 100 vol.% oxygen atmosphere and 4 *F*. After workup, the product is obtained as colorless solid (yield: 19%, 7 mg, 0.05 mmol).

R_f (cyclohexane/ethyl acetate = 1:1 + 1 vol.% glacial acetic acid): 0.40; ^{1}H NMR (400 MHz, DMSO-d_6) δ [ppm] = 12.03 (s, 2H) 2.23–2.18 (m, 4H), 1.54–1.45 (m, 4H); ^{13}C NMR (101 MHz, DMSO-d_6) δ [ppm] = 174.4, 33.4, 24.1; HRMS for $C_6H_9O_4^-$ (ESI–) $[M–H]^-$: calc.: 145.0506, found: 145.0501.

All spectroscopical data match to the reported data[11].

Octane-1,8-dioic acid (6b)

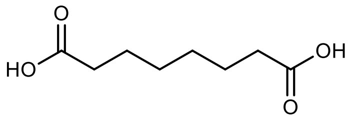

According to the general protocol (GP 1) cyclooctene (28 mg, 0.25 mmol, 1.0 eq.) and tetrabutylammonium nitrate (38 mg, 0.125 mmol, 0.5 eq.) are dissolved in isobutyronitrile (5 mL) and electrolyzed with constant current (5 mA cm^{-2}) at 35 °C, under 100 vol.% oxygen atmosphere and 4 *F*. After workup, the product is obtained as colorless solid (yield: 46%, 20 mg, 0.12 mmol).

R_f (cyclohexane/ethyl acetate = 1:1 + 1 vol.% glacial acetic acid): 0.43; ^{1}H NMR (400 MHz, DMSO-d_6) δ [ppm] = 11.98 (s, 2H), 2.18 (t, J = 7.4 Hz, 4H), 1.51–1.44 (m, 4H), 1.27–1.23 (m, 4H); ^{13}C NMR (101 MHz, DMSO-d_6) δ [ppm] = 174.5, 33.6, 28.3, 24.4; HRMS for $C_8H_{13}O_4^-$ (ESI–) $[M–H]^-$: calc.: 173.0819, found: 173.0815.

All spectroscopical data match to the reported data[12].

Dodecane-1,12-dioic acid (6c)

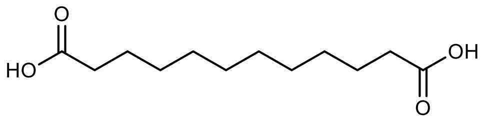

According to the general protocol (GP 1) cyclododecene (42 mg, 0.25 mmol, 1.0 eq.) and tetrabutylammonium nitrate (38 mg, 0.125 mmol, 0.5 eq.) are dissolved in isobutyronitrile (5 mL) and electrolyzed with constant current (5 mA cm^{-2}) at 35 °C, under 100 vol.% oxygen atmosphere and 4 *F*. After workup, the product is obtained as colorless solid (yield: 78%, 45 mg, 0.20 mmol).

R_f (cyclohexane/ethyl acetate = 1:1 + 1 vol.% glacial acetic acid): 0.56; ^{1}H NMR (400 MHz, DMSO-d_6) δ [ppm] = 11.97 (s, 2H), 2.18 (t, J = 7.4 Hz, 4H), 1.51–1.44 (m, 4H), 1.24 (bs, 12H); ^{13}C NMR (101 MHz, DMSO-d_6) δ [ppm] = 174.5, 33.7, 28.9, 28.8, 28.6, 24.5; HRMS for $C_{12}H_{21}O_4^-$ (ESI–) $[M–H]^-$: calc.: 229.1445, found: 229.1439.

All spectroscopical data match to the reported data[11].

Cyclopentane-1,3-dicarboxylic acid (6d)

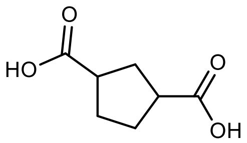

According to the general protocol (GP 1) norbornene (24 mg, 0.25 mmol, 1.0 eq.) and tetrabutylammonium nitrate (38 mg, 0.125 mmol, 0.5 eq.) are dissolved in acetonitrile (5 mL) and electrolyzed with constant current (5 mA cm^{-2}) at 5 °C, under 100 vol.% oxygen atmosphere and 4 *F*. After workup, the product is obtained as off-white highly viscous liquid (yield: 45%, 18 mg, 0.11 mmol).

^{1}H NMR (400 MHz, DMSO-d_6) δ [ppm] = 12.14 (s, 2H), 2.78–2.62 (m, 2H), 2.13–2.06 (m, 1H), 1.88–1.72 (m, 5H); ^{13}C NMR (101 MHz, DMSO-d_6) δ [ppm] = 176.4, 43.3, 32.8, 28.9; HRMS for $C_7H_9O_4^-$ (ESI–) [M–H]$^-$: calc.: 157.0506, found: 157.0507.

All spectroscopical data match to the reported data[13].

6-Oxo-6-phenylhexanoic acid (6e)

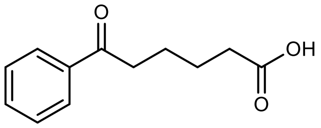

According to the general protocol (GP 1) 1-phenyl-1-cyclohexene (40 mg, 0.25 mmol, 1.0 eq.) and tetrabutylammonium nitrate (38 mg, 0.125 mmol, 0.5 eq.) are dissolved in isobutyronitrile (5 mL) and electrolyzed with constant current (5 mA cm^{-2}) at 20 °C, under 100 vol.% oxygen atmosphere and 4 *F*. After workup, the product is obtained as colorless solid (yield: 26%, 13 mg, 0.07 mmol).

R_f (cyclohexane/ethyl acetate = 1:1 + 1 vol.% glacial acetic acid): 0.60; ^{1}H NMR (400 MHz, DMSO-d_6) δ [ppm] = 12.16 (s, 1H), 7.98–7.94 (m, 2H), 7.65–7.60 (m, 1H), 7.55–7.49 (m, 2H), 3.03 (t, *J* = 7.4 Hz, 2H), 2.25 (t, *J* = 7.4 Hz, 2H), 1.66–1.54 (m, 4H); ^{13}C NMR (101 MHz, DMSO-d_6) δ [ppm] = 199.9, 174.4, 136.7, 133.1, 128.7, 127.9, 37.6, 33.6, 24.1, 23.3; HRMS for $C_{12}H_{13}O_3^-$ (ESI–) [M–H]$^-$: calc.: 205.0870, found: 205.0861.

All spectroscopical data match to the reported data[14].

By-product: 5-Oxo-5-phenylpentanoic acid (**6e'**)

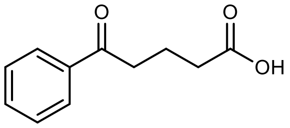

Mol ratio to **6e**: 1:1,9 (yield: 14%, 7 mg, 0.04 mmol).

R_f (cyclohexane/ethyl acetate = 1:1 + 1 vol.% glacial acetic acid): 0.60; ^{1}H NMR (400 MHz, DMSO-d_6) δ [ppm] = 12.16 (s, 1H), 7.98–7.94 (m, 2H), 7.65–7.60 (m, 1H), 7.55–7.49 (m, 2H), 3.06 (t, *J* = 7.4 Hz, 2H), 2.30 (t, *J* = 7.4 Hz, 2H), 1.83 (p, *J* = 7.4 Hz, 2H); ^{13}C NMR (101 MHz, DMSO-d_6) δ [ppm] = 199.6, 174.3, 136.6, 133.1, 128.7, 127.9, 37.2, 32.8, 19.2; HRMS for $C_{11}H_{11}O_3^-$ (ESI–) [M–H]$^-$: calc.: 191.0714, found: 191.0708.

All spectroscopical data match to the reported data[15].

(3*S*)-4-Methyl-3-(3-oxobutyl)pent-4-enoic acid (6f)

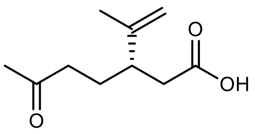

According to the general protocol (GP 1) *S*-(–)-limonene (34 mg, 0.25 mmol, 1.0 eq.) and tetrabutylammonium nitrate (38 mg, 0.125 mmol, 0.5 eq.) are dissolved in acetonitrile (5 mL) and electrolyzed with constant current (5 mA cm^{-2}) at 25 °C, under 100 vol.% oxygen atmosphere and 4 *F*. After workup and column chromatographic purification (cyclohexane/ethyl acetate = 1:1 + 1 vol.% glacial acetic acid), the product is obtained as colorless highly viscous liquid (yield: 17%, 8 mg, 0.04 mmol).

R_f (cyclohexane/ethyl acetate = 1:1 + 1 vol.% glacial acetic acid): 0.53; ^{1}H NMR (400 MHz, DMSO-d_6) δ [ppm] = 12.04 (s, 1H), 4.75–4.69 (m, 2H), 2.44–2.37 (m, 1H), 2.33 (t, *J* = 7.7 Hz, 2H), 2.26 (dd, *J* = 14.9 Hz, *J* = 8.3 Hz, 1H), 2.24 (dd, *J* = 14.9 Hz, *J* = 6.6 Hz, 1H), 2.05 (s, 3H), 1.60 (s, 3H), 1.59–1.45 (m, 2H); ^{13}C NMR (101 MHz, DMSO-d_6) δ [ppm] = 208.1, 173.3, 146.1, 112.0, 42.4, 40.4, 38.6, 29.8, 26.1, 18.6; HRMS for $C_{10}H_{15}O_3^-$ (ESI–) [M–H]$^-$: calc.: 183.1027, found: 183.1029.

Nonane-1,9-dioic acid (9b)

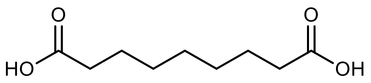

According to the general protocol (GP 1) oleic acid (141 mg, 0.5 mmol, 1.0 eq.) or elaidic acid (141 mg, 0.5 mmol, 1.0 eq.) and tetrabutylammonium nitrate (76 mg, 0.25 mmol, 0.5 eq.) are dissolved in isobutyronitrile (5 mL) and electrolyzed with constant current (10 mA cm^{-2}) at 30 °C, under 100 vol.% oxygen atmosphere and 10 *F*. After workup, and column chromatographic purification (cyclohexane/ethyl acetate = 1:1 + 1 vol.% glacial acetic acid), the product is obtained as colorless solid (from oleic acid: yield: 46%, 44 mg, 0.23 mmol; from elaidic acid: yield: 38%, 36 mg, 0.19 mmol).

R_f (cyclohexane/ethyl acetate = 1:1 + 1 vol.% glacial acetic acid): 0.47; ^{1}H NMR (400 MHz, DMSO-d_6) δ [ppm] = 11.98 (s, 2H), 2.18 (t, *J* = 7.4 Hz, 4H), 1.51–1.43 (m, 4H), 1.25 (bs, 6H); ^{13}C NMR (101 MHz, DMSO-d_6) δ [ppm] = 174.5, 33.7, 28.5, 28.4, 24.5; HRMS for $C_9H_{15}O_4$ (ESI–) [M–H]$^-$: calc.: 187.0976, found: 187.0987.

All spectroscopical data match to the reported data[11].

Benzaldehyde semicarbazone (13a)

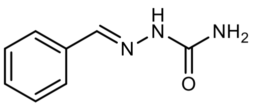

According to the general protocol (GP 3) toluene (46 mg, 0.5 mmol, 1.0 eq.) and tetrabutylammonium nitrate (152 mg, 0.5 mmol, 1.0 eq.) are dissolved in acetonitrile (5 mL) and electrolyzed with constant current (10 mA cm^{-2}) at 33 °C, under 100 vol.% oxygen atmosphere and 5 *F*. After workup and derivatization, the product is obtained as colorless solid (yield: 43%, 35 mg, 0.21 mmol).

m_R: 205–207 °C; R_f (ethyl acetate): 0.44; ^{1}H NMR (400 MHz, DMSO-d_6) δ [ppm] = 10.29 (s, 1H), 7.84 (s, 1H), 7.72–7.69 (m, 2H), 7.40–7.31 (m, 3H), 6.50 (bs, 2H); ^{13}C NMR (101 MHz, DMSO-d_6) δ [ppm] = 156.8, 139.3, 134.8, 129.0, 128.6, 126.6; HRMS for $C_8H_{10}N_3O^+$ (ESI+) $[M+H]^+$: calc.: 164.0818, found: 164.0820.

***o*-Tolualdehyde semicarbazone (13b)**

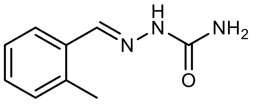

According to the general protocol (GP 3) 1,2-dimethylbenzene (53 mg, 0.5 mmol, 1.0 eq.) and tetrabutylammonium nitrate (152 mg, 0.5 mmol, 1.0 eq.) are dissolved in acetonitrile (5 mL) and electrolyzed with constant current (10 mA cm^{-2}) at 33 °C, under 100 vol.% oxygen atmosphere and 5 *F*. After workup and derivatization, the product is obtained as colorless solid (yield: 58%, 51 mg, 0.29 mmol).

m_R: 199–202 °C; R_f (ethyl acetate): 0.46; ^{1}H NMR (400 MHz, DMSO-d_6) δ [ppm] = 10.21 (s, 1H), 8.14 (s, 1H), 7.93–7.91 (m, 1H), 7.25–7.17 (m, 3H), 6.46 (bs, 2H), 2.36 (s, 3H); ^{13}C NMR (101 MHz, DMSO-d_6) δ [ppm] = 156.7, 137.9, 135.8, 132.6, 130.6, 128.8, 126.0, 125.6, 19.0; HRMS for $C_9H_{12}N_3O^+$ (ESI+) $[M+H]^+$: calc.: 178.0975, found: 178.0978.

All spectroscopical data match to the reported data[16].

***m*-Tolualdehyde semicarbazone (13c)**

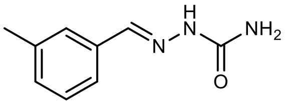

According to the general protocol (GP 3) 1,3-dimethylbenzene (53 mg, 0.5 mmol, 1.0 eq.) and tetrabutylammonium nitrate (152 mg, 0.5 mmol, 1.0 eq.) are dissolved in acetonitrile (5 mL) and electrolyzed with constant current (10 mA cm^{-2}) at 33 °C, under 100 vol.% oxygen atmosphere and 5 *F*. After workup and derivatization, the product is obtained as colorless solid (yield: 54%, 48 mg, 0.27 mmol).

m_R: 206–208 °C; R_f (ethyl acetate): 0.46; ^{1}H NMR (400 MHz, DMSO-d_6) δ [ppm] = 10.24 (s, 1H), 7.80 (s, 1H), 7.56 (s, 1H), 7.47 (d, *J* = 7.6 Hz, 1H), 7.26 (t, *J* = 7.6 Hz, 1H), 7.15 (d, *J* = 7.6 Hz, 1H), 6.50 (bs, 2H), 2.31 (s, 3H); ^{13}C NMR (101 MHz, DMSO-d_6) δ [ppm] = 156.8, 139.4, 137.8, 134.7, 129.7, 128.5, 126.9, 124.0, 20.9; HRMS for $C_9H_{12}N_3O^+$ (ESI+) $[M+H]^+$: calc.: 178.0975, found: 178.0975.

p-Tolualdehyde semicarbazone (13d)

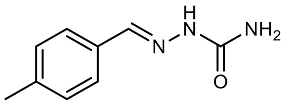

According to the general protocol (GP 3) 1,4-dimethylbenzene (53 mg, 0.5 mmol, 1.0 eq.) and tetrabutylammonium nitrate (152 mg, 0.5 mmol, 1.0 eq.) are dissolved in acetonitrile (5 mL) and electrolyzed with constant current (10 mA cm^{-2}) at 33 °C, under 100 vol.% oxygen atmosphere and 5 *F*. After workup and derivatization, the product is obtained as colorless solid (yield: 68%, 60 mg, 0.34 mmol).

m_R: 203–206 °C; R_f (ethyl acetate): 0.44; ^{1}H NMR (400 MHz, DMSO-d_6) δ [ppm] = 10.19 (s, 1H), 7.80 (s, 1H), 7.60 (d, J = 8.0 Hz, 2H), 7.19 (d, J = 8.0 Hz, 2H), 6.46 (bs, 2H), 2.31 (s, 3H); ^{13}C NMR (101 MHz, DMSO-d_6) δ [ppm] = 156.8, 139.3, 138.6, 132.1, 129.2, 126.5, 21.0; HRMS for $C_9H_{12}N_3O^+$ (ESI+) $[M+H]^+$: calc.: 178.0975, found: 178.0975.

All spectroscopical data match to the reported data[17].

3,5-Dimethylbenzaldehyde semicarbazone (13e)

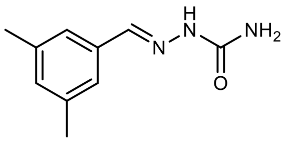

According to the general protocol (GP 3) 1,3,5-trimethylbenzene (60 mg, 0.5 mmol, 1.0 eq.) and tetrabutylammonium nitrate (152 mg, 0.5 mmol, 1.0 eq.) are dissolved in acetonitrile (5 mL) and electrolyzed with constant current (10 mA cm^{-2}) at 33 °C, under 100 vol.% oxygen atmosphere and 5 *F*. After workup and derivatization, the product is obtained as colorless solid (yield: 68%, 65 mg, 0.34 mmol).

m_R: 200–202 °C; R_f (ethyl acetate): 0.41; ^{1}H NMR (400 MHz, DMSO-d_6) δ [ppm] = 10.20 (s, 1H), 7.76 (s, 1H), 7.32 (s, 2H), 6.96 (s, 1H), 6.48 (bs, 2H), 2.27 (s, 6H); ^{13}C NMR (101 MHz, DMSO-d_6) δ [ppm] = 156.8, 139.5, 137.6, 134.7, 130.6, 124.4, 20.8; HRMS for $C_{10}H_{14}N_3O^+$ (ESI+) $[M+H]^+$: calc.: 192.1131, found: 192.1132.

4-Fluorobenzaldehyde semicarbazone (13f)

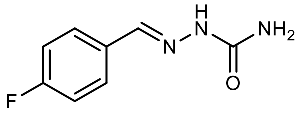

According to the general protocol (GP 3) 4-fluorotoluene (55 mg, 0.5 mmol, 1.0 eq.) and tetrabutylammonium nitrate (152 mg, 0.5 mmol, 1.0 eq.) are dissolved in acetonitrile (5 mL) and electrolyzed with constant current (10 mA cm^{-2}) at 33 °C, under 100 vol.% oxygen atmosphere and 5 *F*. After workup and derivatization, the product is obtained as colorless solid (yield: 21%, 19 mg, 0.10 mmol).

m_R: 204–207 °C; R_f (ethyl acetate): 0.39; ^{1}H NMR (400 MHz, DMSO-d_6) δ [ppm] = 10.26 (s, 1H), 7.82 (s, 1H), 7.81–7.76 (m, 2H), 7.24–7.18 (m, 2H), 6.51 (bs, 2H); ^{13}C NMR (101 MHz, DMSO-d_6) δ [ppm] = 162.5 (d, J = 246.4 Hz), 156.8, 138.0, 131.5 (d, J = 3.0 Hz), 128.6 (d, J = 8.3 Hz), 115.6 (d, J = 21.7 Hz); ^{19}F NMR (282 MHz, DMSO-d_6) δ [ppm] = –112.23; HRMS for $C_8H_9FN_3O^+$ (ESI+) $[M+H]^+$: calc.: 182.0724, found: 182.0728.

4-Chlorobenzaldehyde semicarbazone (13g)

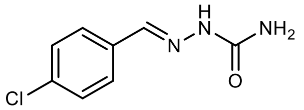

According to the general protocol (GP 3) 4-chlorotoluene (63 mg, 0.5 mmol, 1.0 eq.) and tetrabutylammonium nitrate (152 mg, 0.5 mmol, 1.0 eq.) are dissolved in acetonitrile (5 mL) and electrolyzed with constant current (10 mA cm^{-2}) at 33 °C, under 100 vol.% oxygen atmosphere and 5 *F*. After workup and derivatization, the product is obtained as colorless solid (yield: 35%, 35 mg, 0.18 mmol).

m_R: 208–212 °C; R_f (ethyl acetate): 0.39; ^{1}H NMR (400 MHz, DMSO-d_6) δ [ppm] = 10.33 (s, 1H), 7.81 (s, 1H), 7.77–7.74 (m, 2H), 7.44–7.41 (m, 2H), 6.55 (bs, 2H); ^{13}C NMR (101 MHz, DMSO-d_6) δ [ppm] = 156.7, 137.9, 133.8, 133.4, 128.6, 128.2; HRMS for $C_8H_9{}^{35}ClN_3O^+$ (ESI+) $[M+H]^+$: calc.: 198.0429, found: 198.0430.

All spectroscopical data match to the reported data[17].

Benzoic acid (14a)

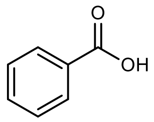

According to the general protocol (GP 3) toluene (230 mg, 2.5 mmol, 1.0 eq.) and tetrabutylammonium nitrate (381 mg, 1.25 mmol, 0.5 eq.) are dissolved in acetonitrile (25 mL) and electrolyzed with constant current (30 mA cm^{-2}) at 27 °C, under 100 vol.% oxygen atmosphere and 12 *F*. After workup and column chromatographic purification (cyclohexane/ethyl acetate = 1:1 + 1 vol.% glacial acetic acid), the product is obtained as colorless solid (yield: 37%, 112 mg, 0.92 mmol).

m_R: 117–120 °C; R_f (cyclohexane/ethyl acetate = 1:1 + 1 vol.% glacial acetic acid): 0.67; ^{1}H NMR (400 MHz, DMSO-d_6) δ [ppm] = 12.95 (s, 1H), 7.97–7.94 (m, 2H), 7.64–7.59 (m, 1H), 7.52–7.47 (m, 2H); ^{13}C NMR (101 MHz, DMSO-d_6) δ [ppm] = 167.4, 132.9, 130.8, 129.3, 128.6; HRMS for $C_7H_5O_2^-$ (ESI–) [M–H]$^-$: calc.: 121.0295, found: 121.0292.

All spectroscopical data match to the reported data[18].

2-Methylbenzoic acid (14b)

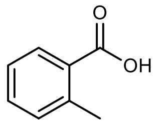

According to the general protocol (GP 3) 1,2-dimethylbenzene (265 mg, 2.5 mmol, 1.0 eq.) and tetrabutylammonium nitrate (381 mg, 1.25 mmol, 0.5 eq.) are dissolved in acetonitrile (25 mL) and electrolyzed with constant current (30 mA cm^{-2}) at 27 °C, under 100 vol.% oxygen atmosphere and 12 *F*. After workup and column chromatographic purification (cyclohexane/ethyl acetate = 1:1 + 1 vol.% glacial acetic acid), the product is obtained as colorless solid (yield: 21%, 72 mg, 0.53 mmol).

m_R: 99–101 °C; R_f (cyclohexane/ethyl acetate = 1:1 + 1 vol.% glacial acetic acid): 0.66; ^{1}H NMR (400 MHz, DMSO-d_6) δ [ppm] = 12.81 (s, 1H), 7.82–7.80 (m, 1H), 7.46–7.42 (m, 1H), 7.30–7.25 (m, 2H), 2.51 (s, 3H); ^{13}C NMR (101 MHz, DMSO-d_6) δ [ppm] = 168.7, 139.0, 131.8, 131.5, 130.4, 130.2, 125.9, 21.3; HRMS for $C_8H_7O_2^-$ (ESI–) [M–H]$^-$: calc.: 135.0452, found: 135.0447.

All spectroscopical data match to the reported data[18].

3-Methylbenzoic acid (14c)

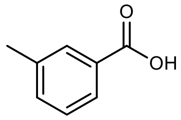

According to the general protocol (GP 3) 1,3-dimethylbenzene (265 mg, 2.5 mmol, 1.0 eq.) and tetrabutylammonium nitrate (381 mg, 1.25 mmol, 0.5 eq.) are dissolved in acetonitrile (25 mL) and electrolyzed with constant current (30 mA cm^{-2}) at 27 °C, under 100 vol.% oxygen atmosphere and 12 *F*. After workup and column chromatographic purification (cyclohexane/ethyl acetate = 1:1 + 1 vol.% glacial acetic acid), the product is obtained as colorless solid (yield: 14%, 48 mg, 0.35 mmol).

m_R: 96–100 °C; R_f (cyclohexane/ethyl acetate = 1:1 + 1 vol.% glacial acetic acid): 0.67; ^{1}H NMR (400 MHz, DMSO-d_6) δ [ppm] = 12.89 (s, 1H), 7.77–7.72 (m, 2H), 7.44–7.41 (m, 1H), 7.39–7.35 (m, 1H), 2.35 (s, 3H); ^{13}C NMR (101 MHz, DMSO-d_6) δ [ppm] = 167.4, 137.9, 133.5, 130.7, 129.8, 128.5, 126.5, 20.8; HRMS for $C_8H_7O_2^-$ (ESI–) [M–H]$^-$: calc.: 135.0452, found: 135.0449.

All spectroscopical data match to the reported data[18].

4-Methylbenzoic acid (14d)

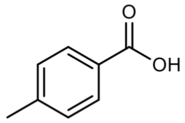

According to the general protocol (GP 3) 1,4-dimethylbenzene (265 mg, 2.5 mmol, 1.0 eq.) and tetrabutylammonium nitrate (381 mg, 1.25 mmol, 0.5 eq.) are dissolved in acetonitrile (25 mL) and electrolyzed with constant current (30 mA cm^{-2}) at 27 °C, under 100 vol.% oxygen atmosphere and 12 *F*. After workup and column chromatographic purification (cyclohexane/ethyl acetate = 1:1 + 1 vol.% glacial acetic acid), the product is obtained as colorless solid (yield: 38%, 128 mg, 0.94 mmol).

m_R: 174–178 °C; R_f (cyclohexane/ethyl acetate = 1:1 + 1 vol.% glacial acetic acid): 0.67; ^{1}H NMR (400 MHz, DMSO-d_6) δ [ppm] = 12.79 (s, 1H), 7.83 (d, J = 8.1 Hz, 2H), 7.29 (d, J = 8.1 Hz, 2H), 2.36 (s, 3H); ^{13}C NMR (101 MHz, DMSO-d_6) δ [ppm] = 167.4, 143.1, 129.4, 129.2, 128.1, 21.2; HRMS for $C_8H_7O_2^-$ (ESI–) [M–H]$^-$: calc.: 135.0452, found: 135.0449.

All spectroscopical data match to the reported data[18].

3,5-Dimethylbenzoic acid (14e)

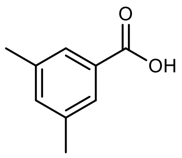

According to the general protocol (GP 3) 1,3,5-trimethylbenzene (301 mg, 2.5 mmol, 1.0 eq.) and tetrabutylammonium nitrate (381 mg, 1.25 mmol, 0.5 eq.) are dissolved in acetonitrile (25 mL) and electrolyzed with constant current (30 mA cm^{-2}) at 27 °C, under 100 vol.% oxygen atmosphere and 7 F. After workup and column chromatographic purification (cyclohexane/ethyl acetate = 1:1 + 1 vol.% glacial acetic acid), the product is obtained as colorless solid (yield: 17%, 62 mg, 0.41 mmol).

m_R: 154–157 °C; R_f (cyclohexane/ethyl acetate = 1:1 + 1 vol.% glacial acetic acid): 0.68; ^{1}H NMR (400 MHz, DMSO-d_6) δ [ppm] = 12.78 (s, 1H), 7.55 (s, 2H), 7.22 (s, 1H), 2.31 (s, 3H); ^{13}C NMR (101 MHz, DMSO-d_6) δ [ppm] = 167.5, 137.7, 134.2, 130.7, 127.0, 20.7; HRMS for $C_9H_9O_2^-$ (ESI–) [M–H]$^-$: calc.: 149.0608, found: 149.0618.

All spectroscopical data match to the reported data[18].

4-Fluorobenzoic acid (14f)

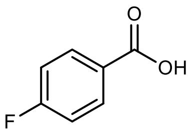

According to the general protocol (GP 3) 4-fluorotoluene (275 mg, 2.5 mmol, 1.0 eq.) and tetrabutylammonium nitrate (381 mg, 1.25 mmol, 0.5 eq.) are dissolved in acetonitrile (25 mL) and electrolyzed with constant current (30 mA cm^{-2}) at 27 °C, under 100 vol.% oxygen atmosphere and 12 F. After workup and column chromatographic purification (cyclohexane/ethyl acetate = 1:1 + 1 vol.% glacial acetic acid), the product is obtained as colorless solid (yield: 36%, 127 mg, 0.91 mmol).

m_R: 182–184 °C; R_f (cyclohexane/ethyl acetate = 1:1 + 1 vol.% glacial acetic acid): 0.62; ^{1}H NMR (400 MHz, DMSO-d_6) δ [ppm] = 13.05 (s, 1H), 8.02–7.97 (m, 2H), 7.34–7.28 (m, 2H); ^{13}C NMR (101 MHz, DMSO-d_6) δ [ppm] = 166.4, 164.9 (d, J = 250.6 Hz), 132.1 (d, J = 9.6 Hz), 127.4 (d, J = 2.8 Hz), 115.6 (d, J = 22.1 Hz); ^{19}F NMR (376 MHz, DMSO-d_6) δ [ppm] = –108.08; HRMS for $C_7H_4FO_2^-$ (ESI–) [M–H]$^-$: calc.: 139.0201, found: 139.0199.

All spectroscopical data match to the reported data[18].

4-Chlorobenzoic acid (14g)

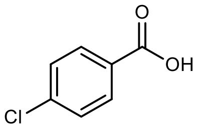

According to the general protocol (GP 3) 4-chlorotoluene (316 mg, 2.5 mmol, 1.0 eq.) and tetrabutylammonium nitrate (381 mg, 1.25 mmol, 0.5 eq.) are dissolved in acetonitrile (25 mL) and electrolyzed with constant current (30 mA cm^{-2}) at 27 °C, under 100 vol.% oxygen atmosphere and 12 F. After workup and column chromatographic purification (cyclohexane/ethyl acetate = 1:1 + 1 vol.% glacial acetic acid), the product is obtained as colorless solid (yield: 46%, 180 mg, 1.15 mmol).

m_R: 229–234 °C; R_f (cyclohexane/ethyl acetate = 1:1 + 1 vol.% glacial acetic acid): 0.57; ^{1}H NMR (400 MHz, DMSO-d_6) δ [ppm] = 13.18 (s, 1H), 7.95–7.92 (m, 2H), 7.58–7.54 (m, 2H); ^{13}C NMR (101 MHz, DMSO-d_6) δ [ppm] = 166.5, 137.8, 131.2, 129.7, 128.8; HRMS for $C_7H_4ClO_2^-$ (ESI–) [M–H]$^-$: calc.: 154.9905, found: 154.9905.

All spectroscopical data match to the reported data[18].

4. Supplementary Spectra (literature unreported)

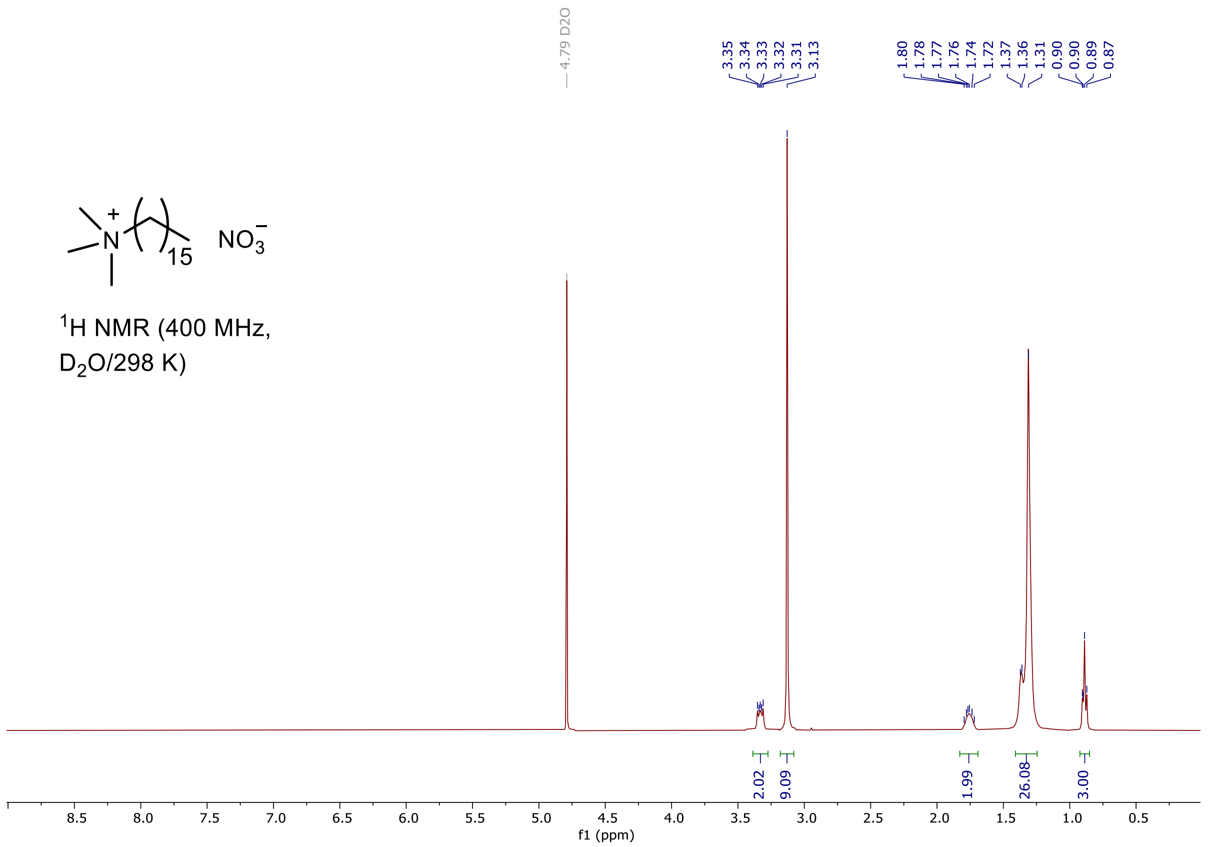

Supplementary Fig. 20: ^{1}H NMR (400 MHz, D_2O, 298 K) of hexadecyltrimethylammonium nitrate.

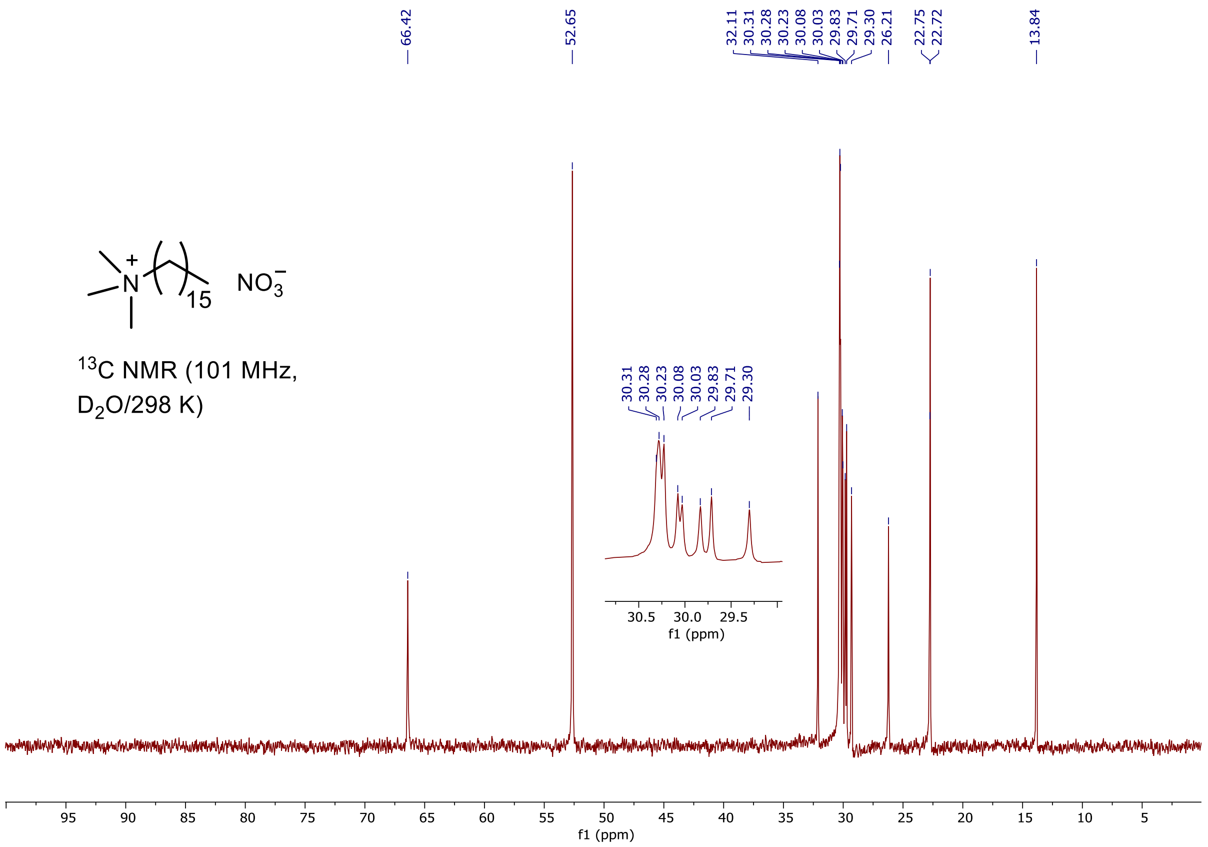

Supplementary Fig. 21: ^{13}C NMR (101 MHz, D_2O, 298 K) of hexadecyltrimethylammonium nitrate.

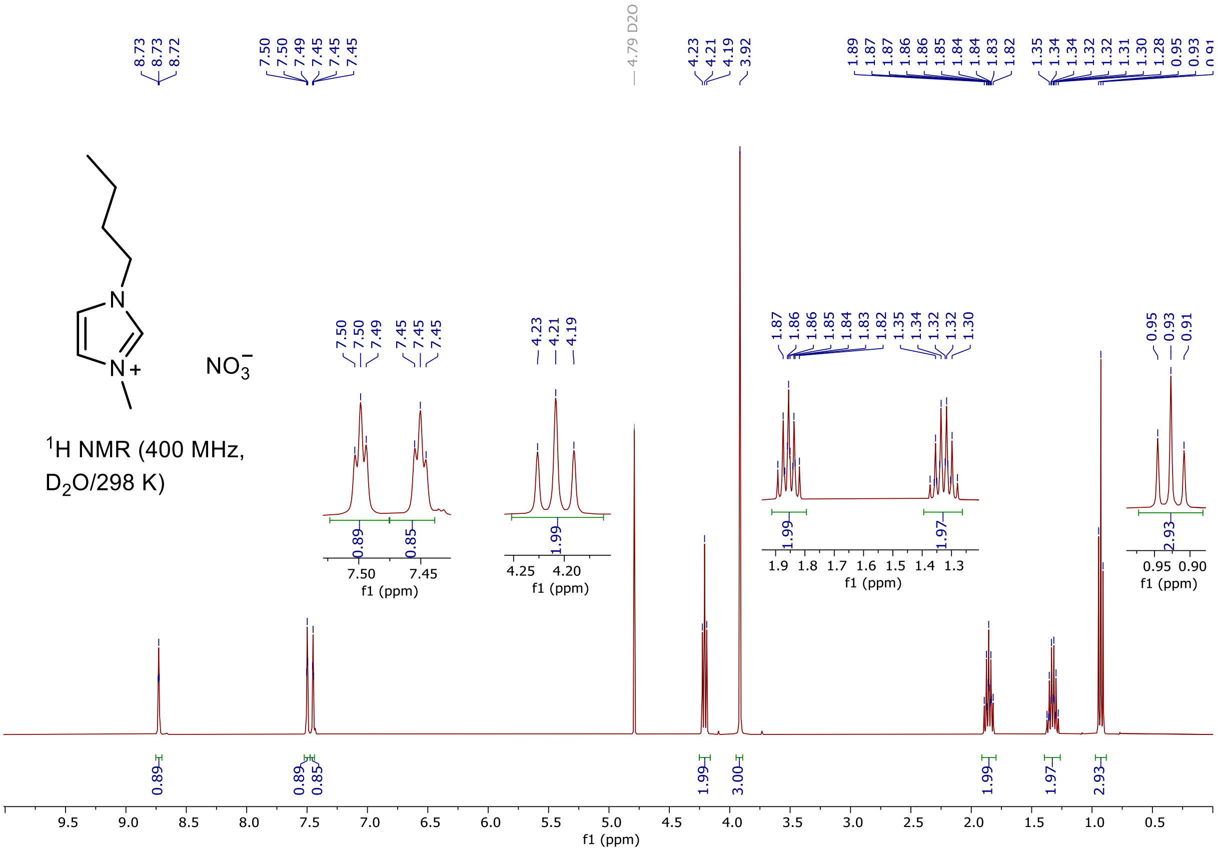

Supplementary Fig. 22: ^{1}H NMR (400 MHz, D_2O, 298 K) of 1-butyl-3-methylimidazolium nitrate.

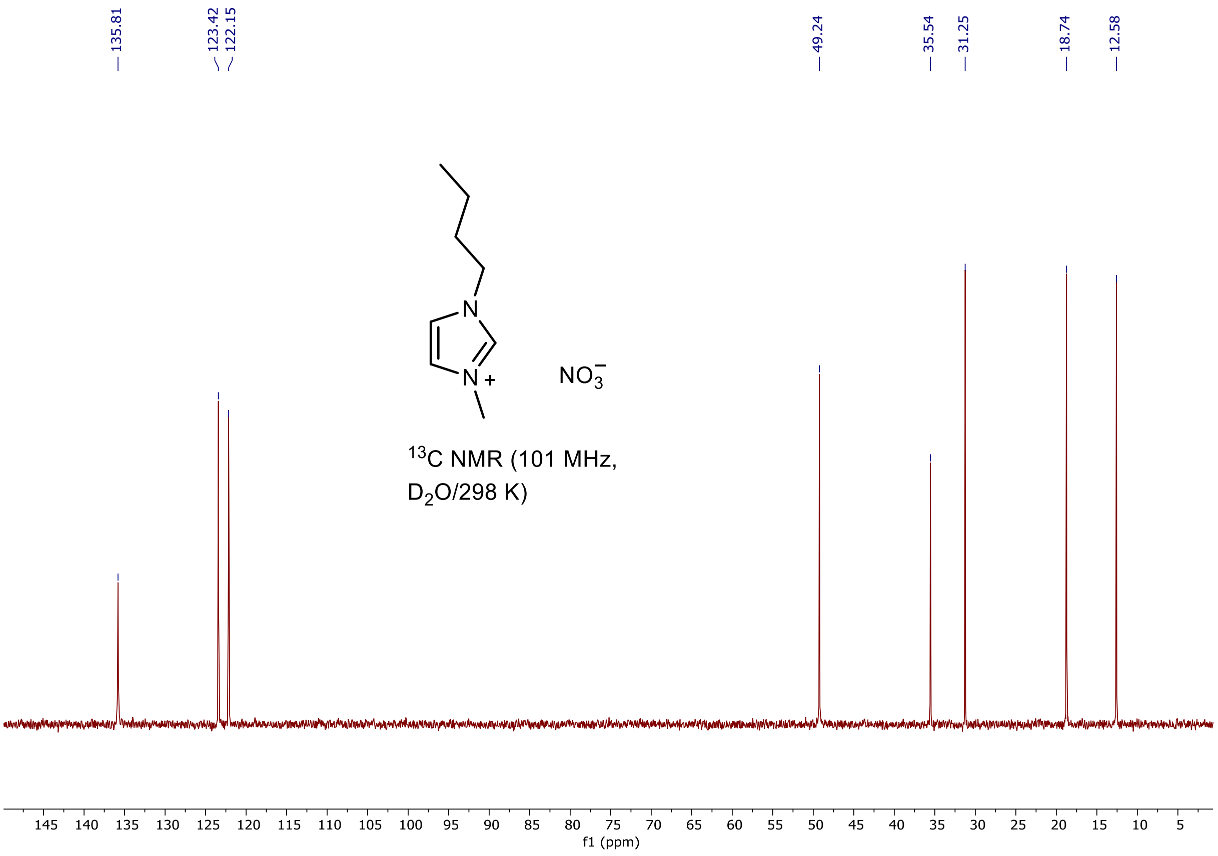

Supplementary Fig. 23: ^{13}C NMR (101 MHz, D_2O, 298 K) of 1-butyl-3-methylimidazolium nitrate.

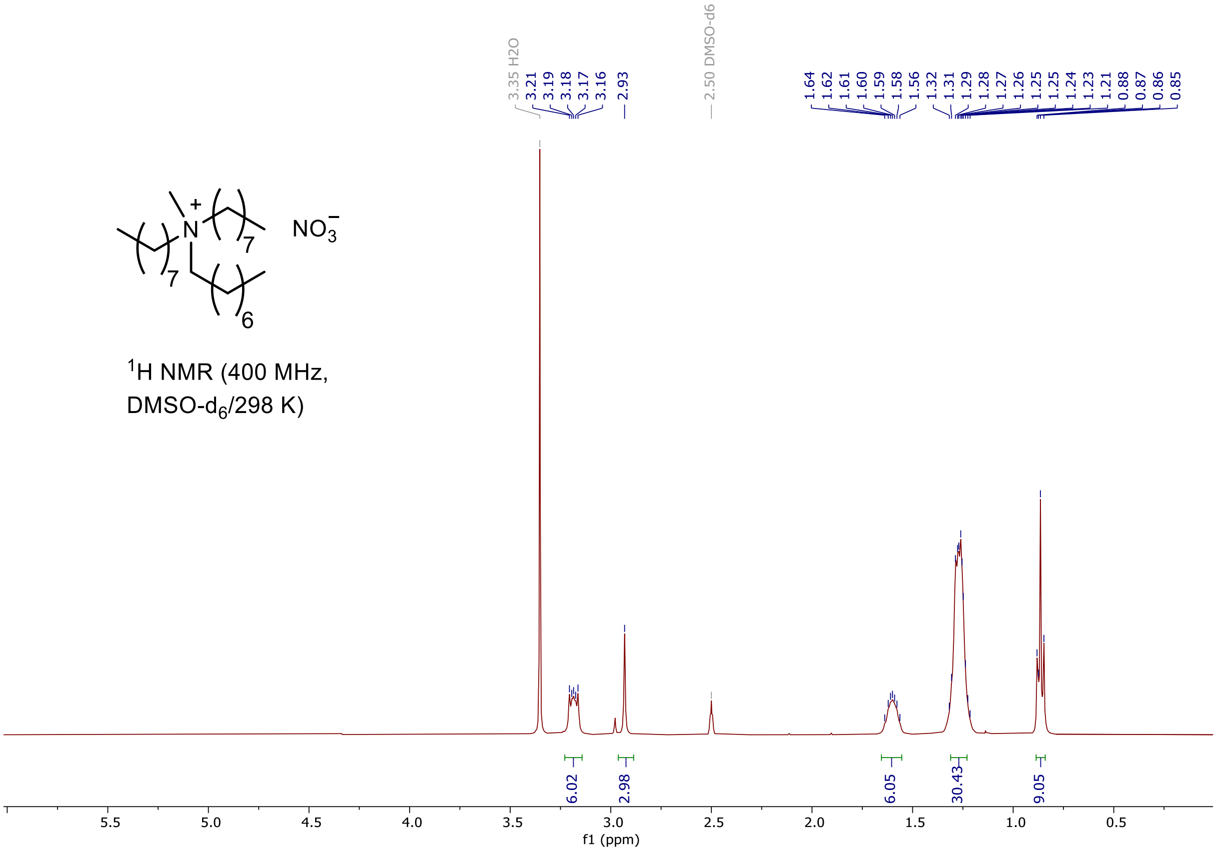

Supplementary Fig. 24: ^{1}H NMR (400 MHz, DMSO-d_6, 298 K) of methyltrioctylammonium nitrate.

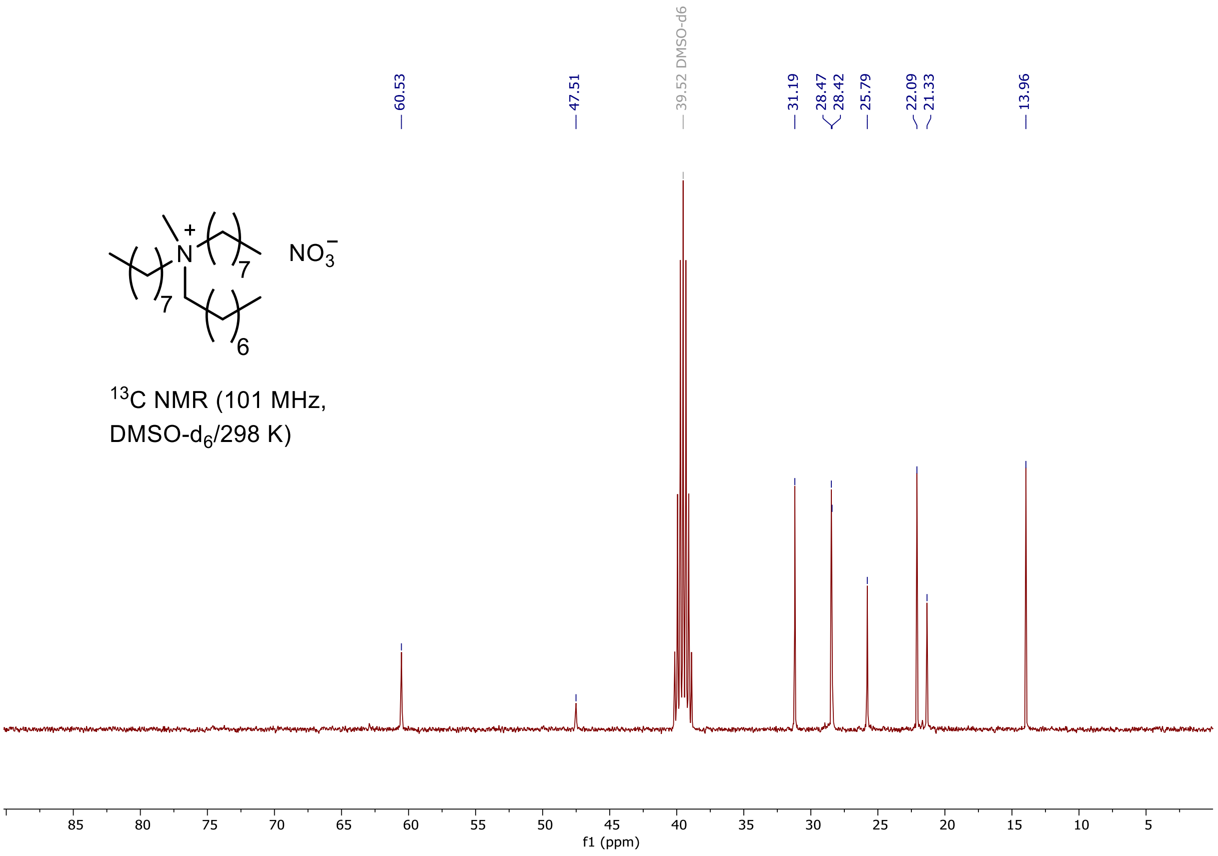

Supplementary Fig. 25: ^{13}C NMR (101 MHz, DMSO-d_6, 298 K) of methyltrioctylammonium nitrate.

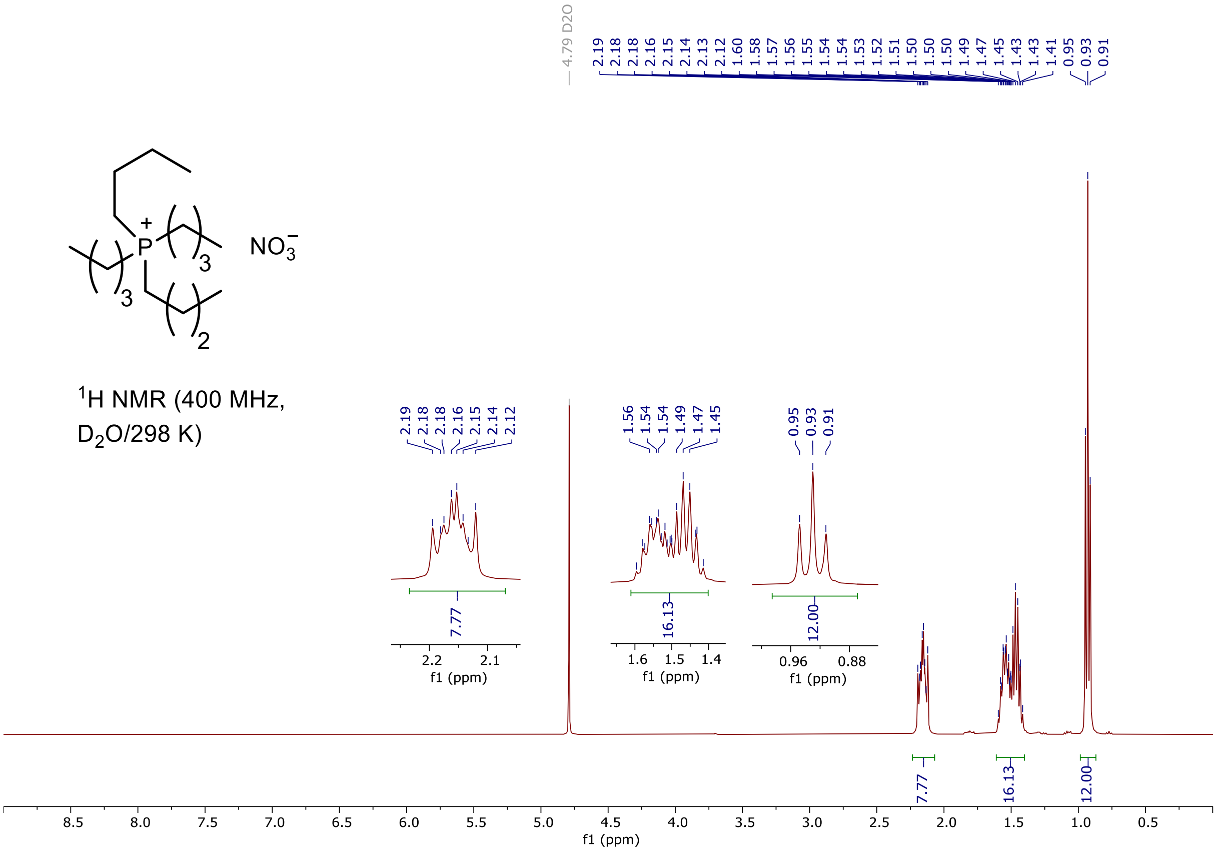

Supplementary Fig. 26: ^{1}H NMR (400 MHz, D_2O, 298 K) of tetrabutylphosphonium nitrate.

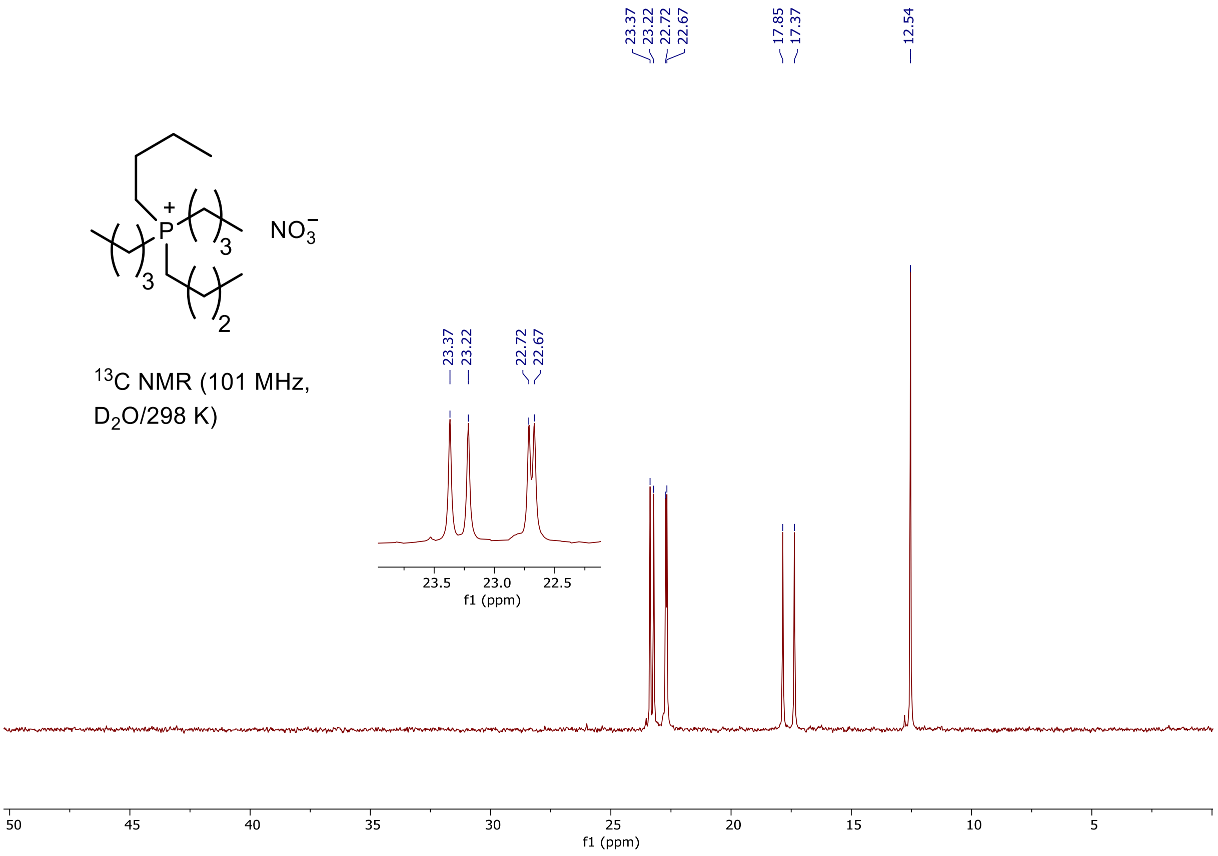

Supplementary Fig. 27: ^{13}C NMR (101 MHz, D_2O, 298 K) of tetrabutylphosphonium nitrate.

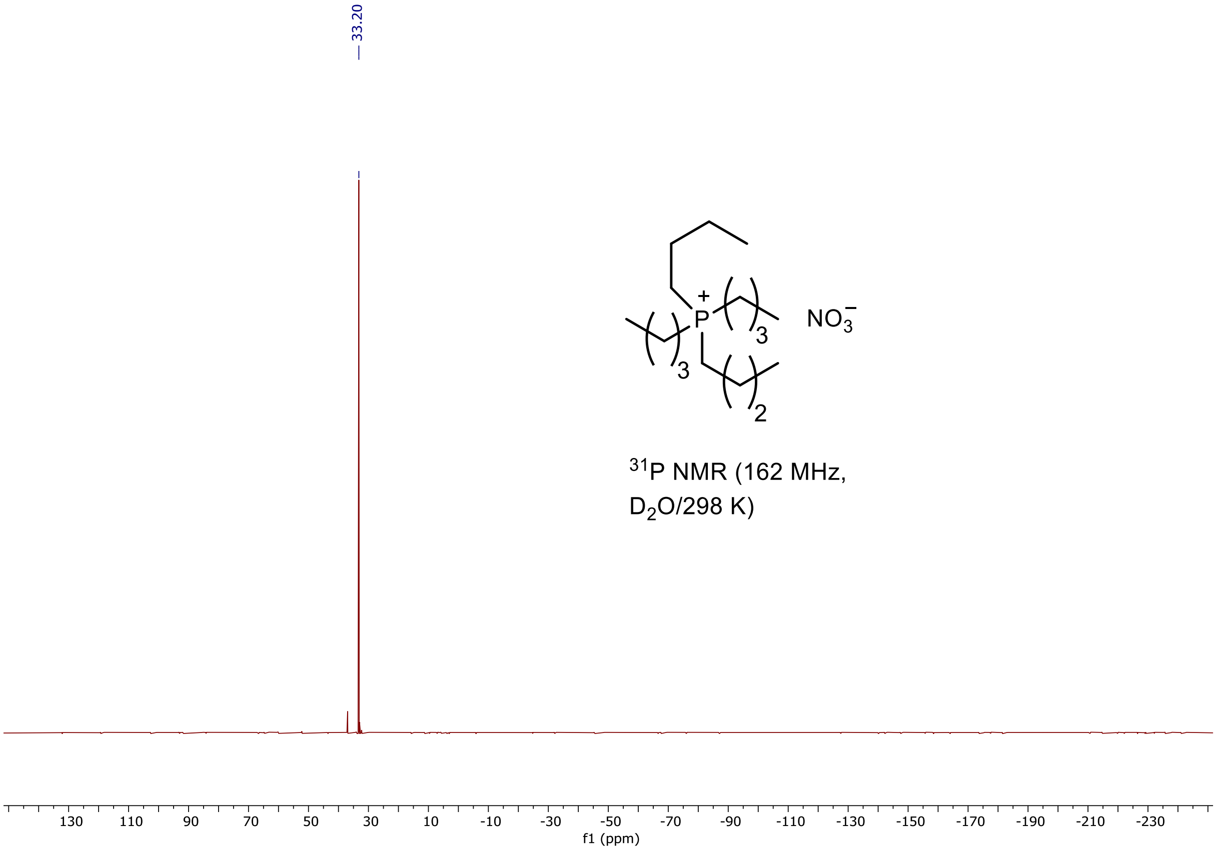

Supplementary Fig. 28: ^{31}P NMR (162 MHz, D_2O, 298 K) of tetrabutylphosphonium nitrate.

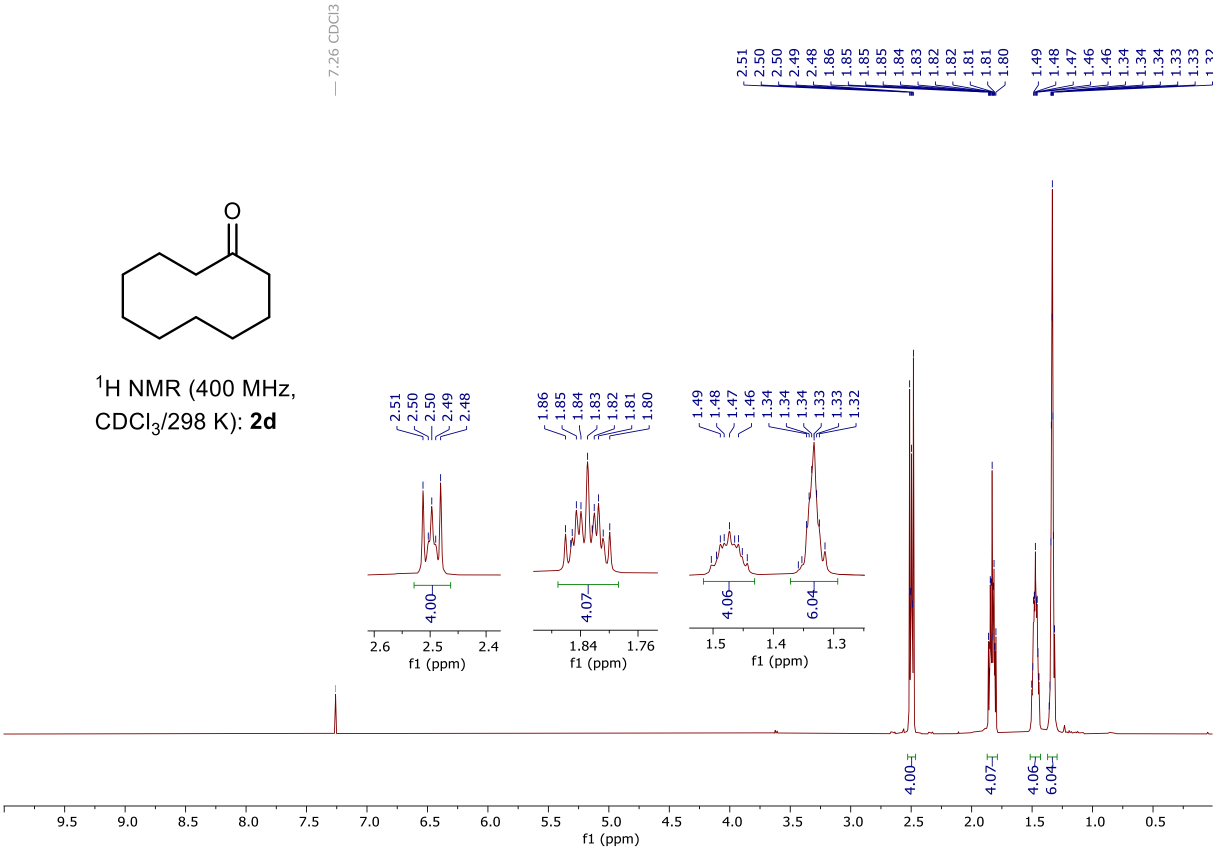

Supplementary Fig. 29: ^{1}H NMR (400 MHz, $CDCl_3$, 298 K) of compound **2d**.

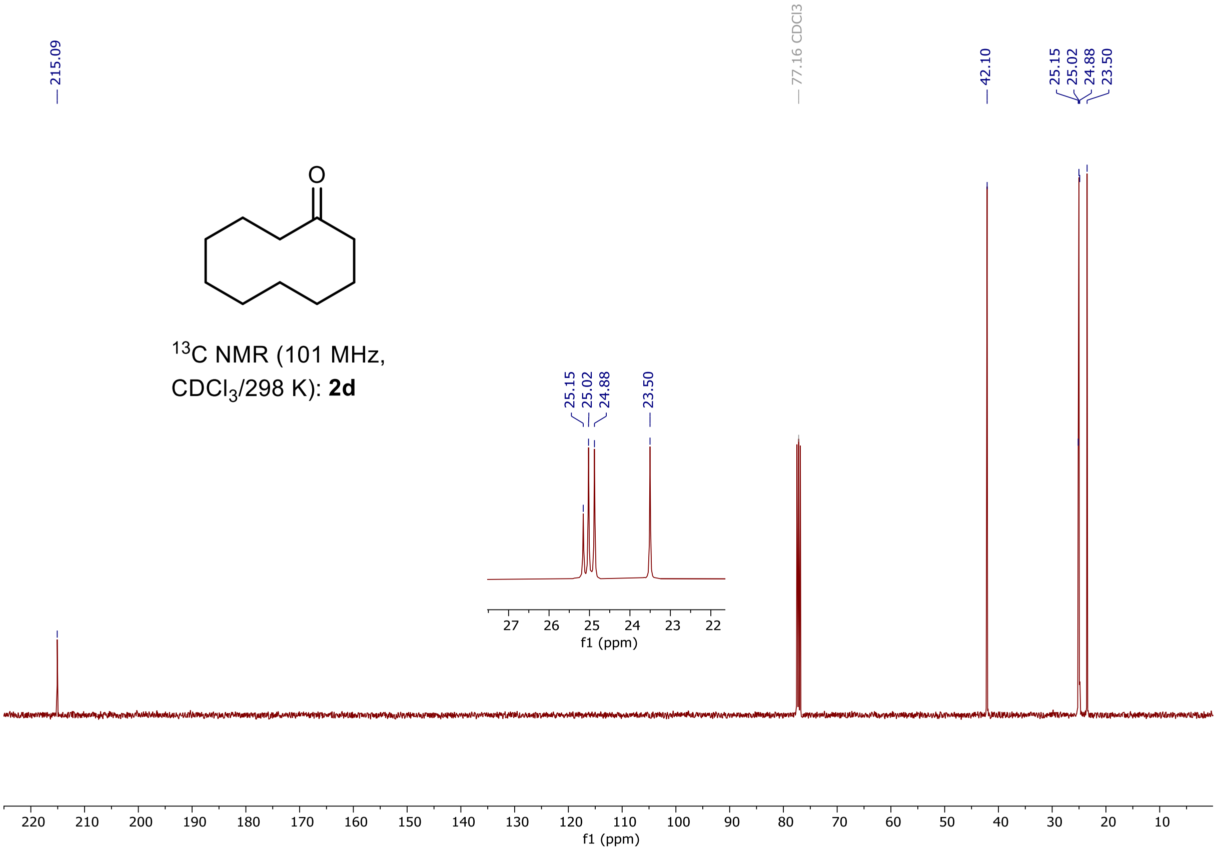

Supplementary Fig. 30: ^{13}C NMR (101 MHz, $CDCl_3$, 298 K) of compound **2d**.

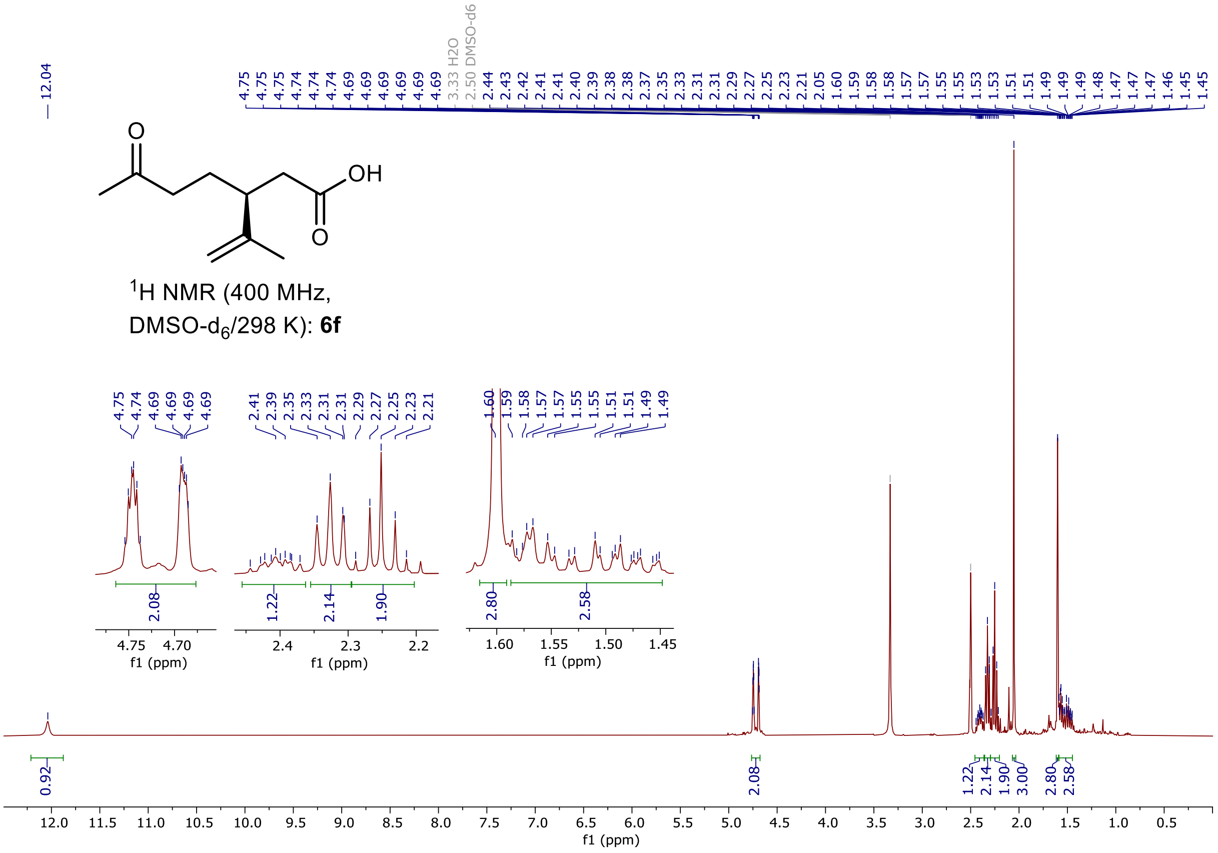

Supplementary Fig. 31: ^{1}H NMR (400 MHz, DMSO-d_6, 298 K) of compound **6f**.

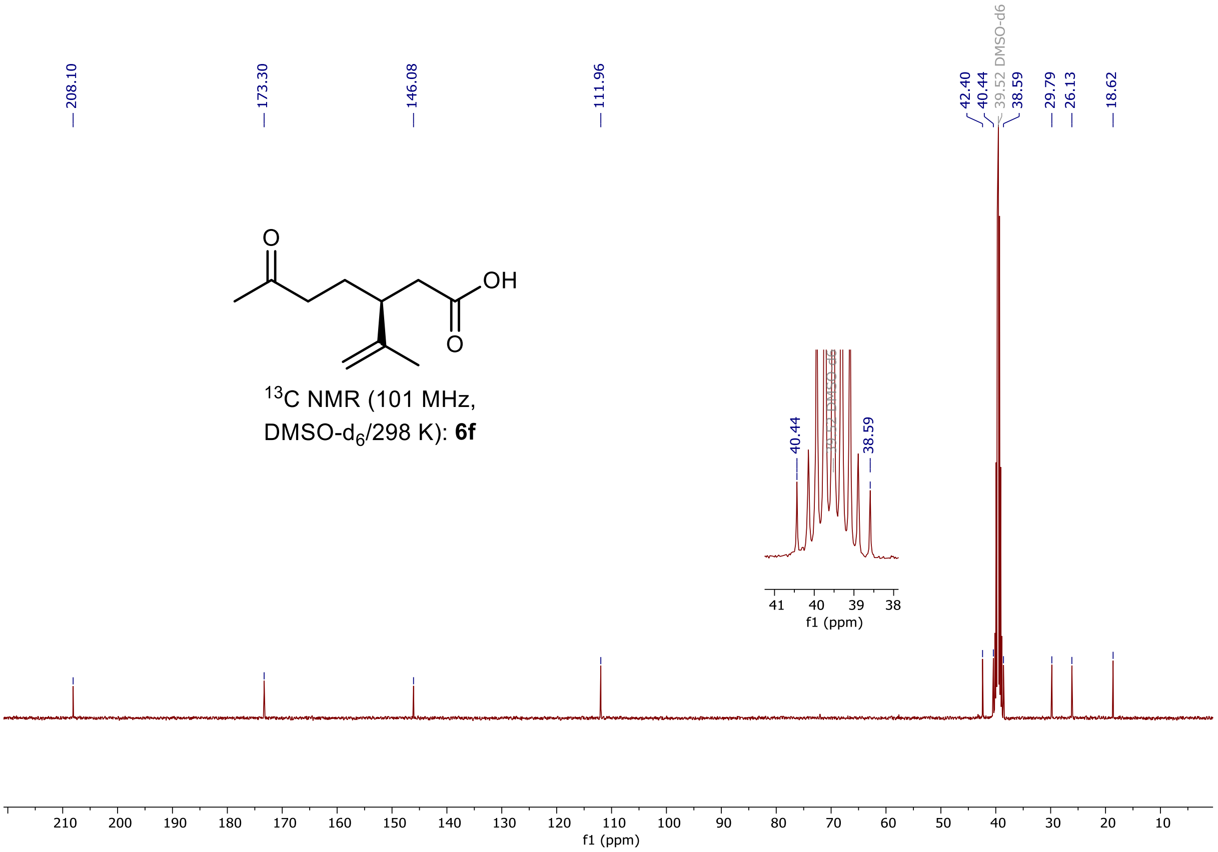

Supplementary Fig. 32: ^{13}C NMR (101 MHz, DMSO-d$_6$, 298 K) of compound **6f**.

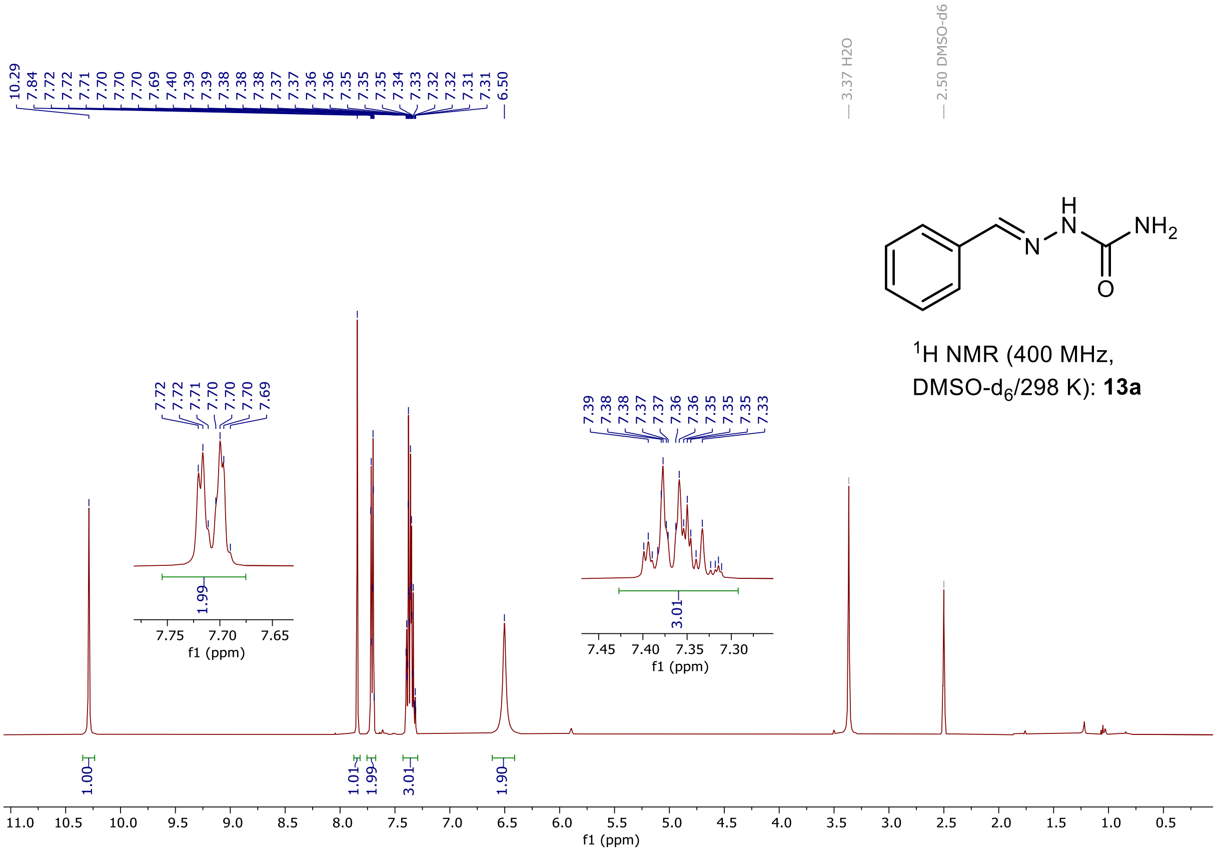

Supplementary Fig. 33: ^{1}H NMR (400 MHz, DMSO-d$_6$, 298 K) of compound **13a**.

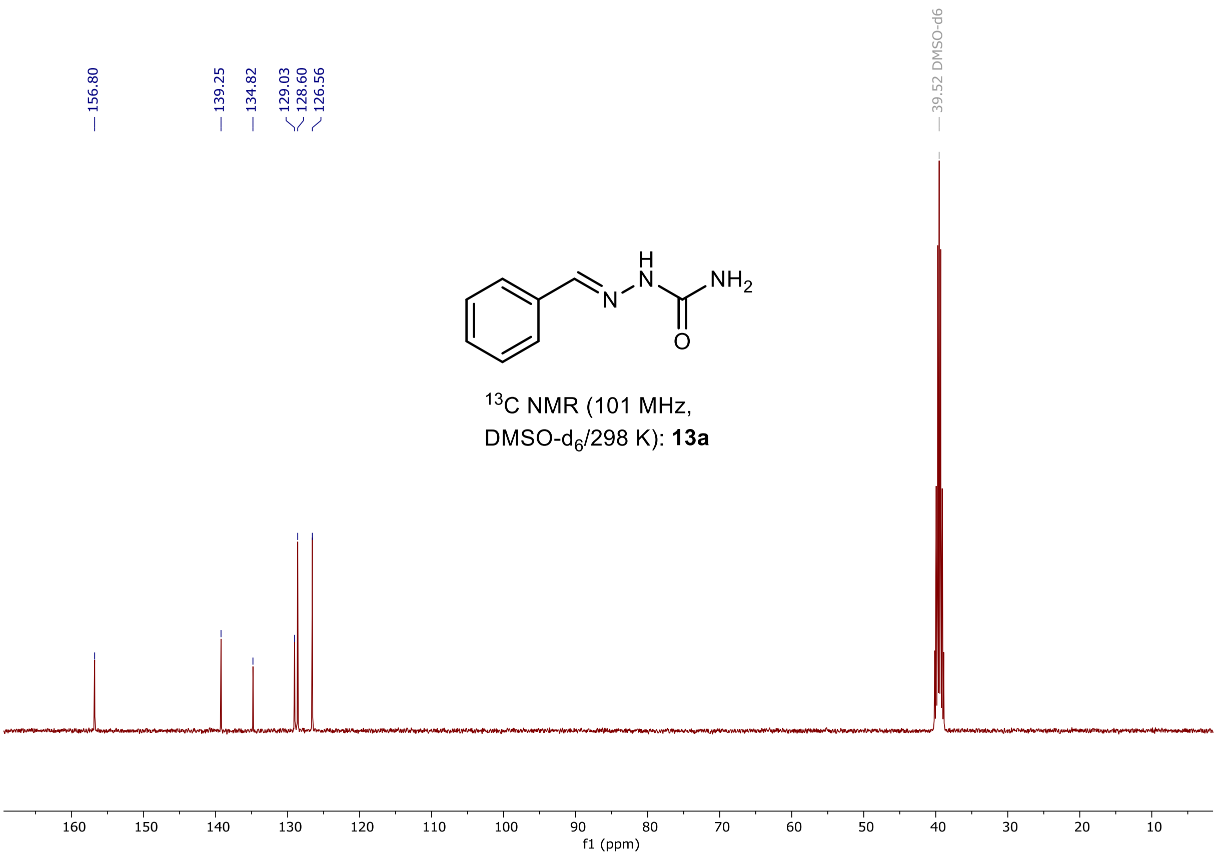

Supplementary Fig. 34: ^{13}C NMR (101 MHz, DMSO-d$_6$, 298 K) of compound **13a**.

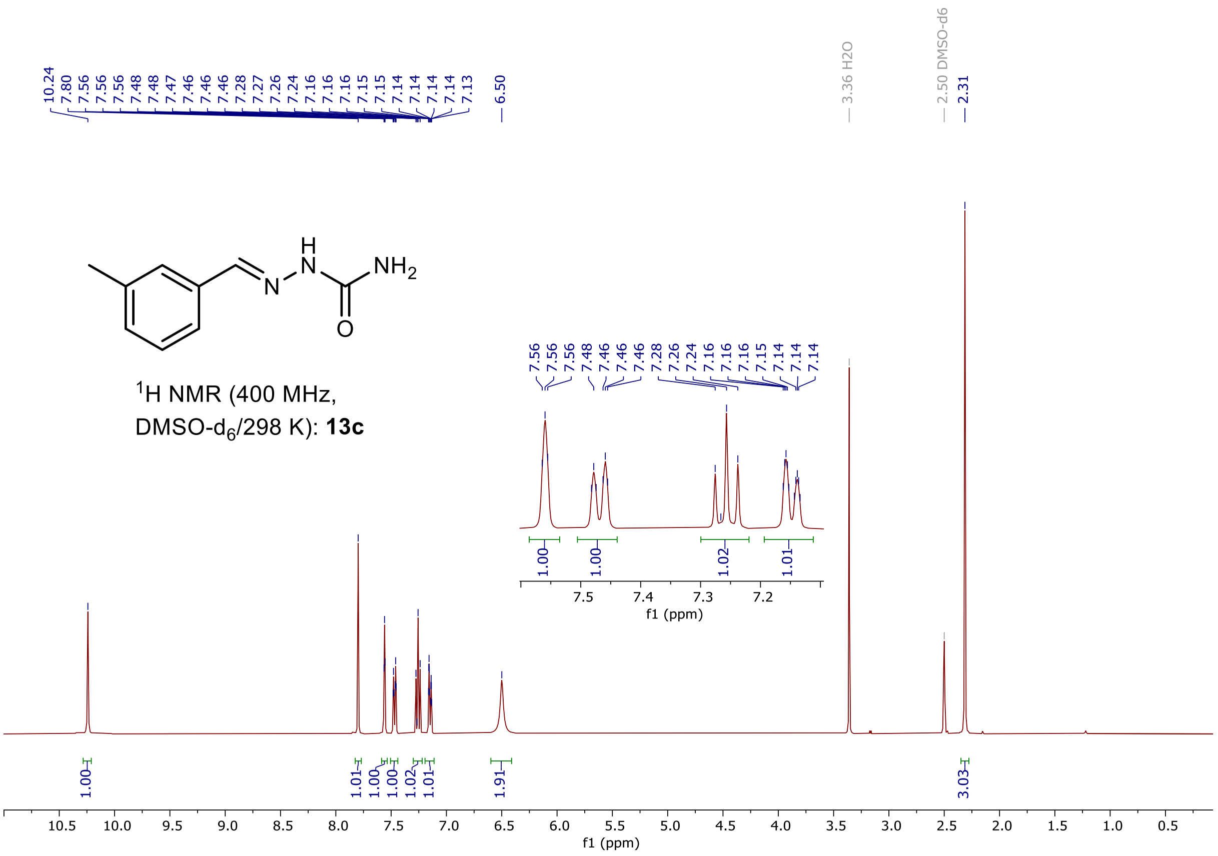

Supplementary Fig. 35: ^{1}H NMR (400 MHz, DMSO-d$_6$, 298 K) of compound **13c**.

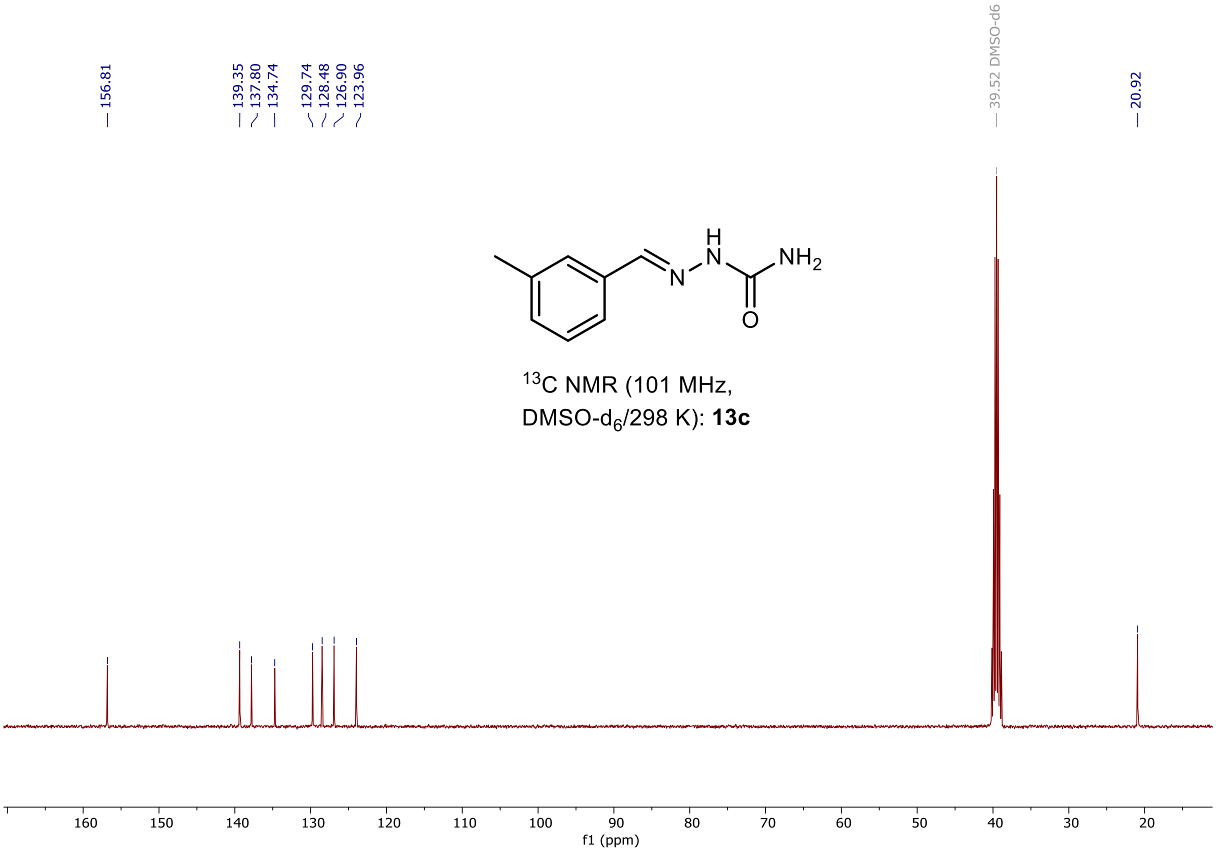

Supplementary Fig. 36: ^{13}C NMR (101 MHz, DMSO-d$_6$, 298 K) of compound **13c**.

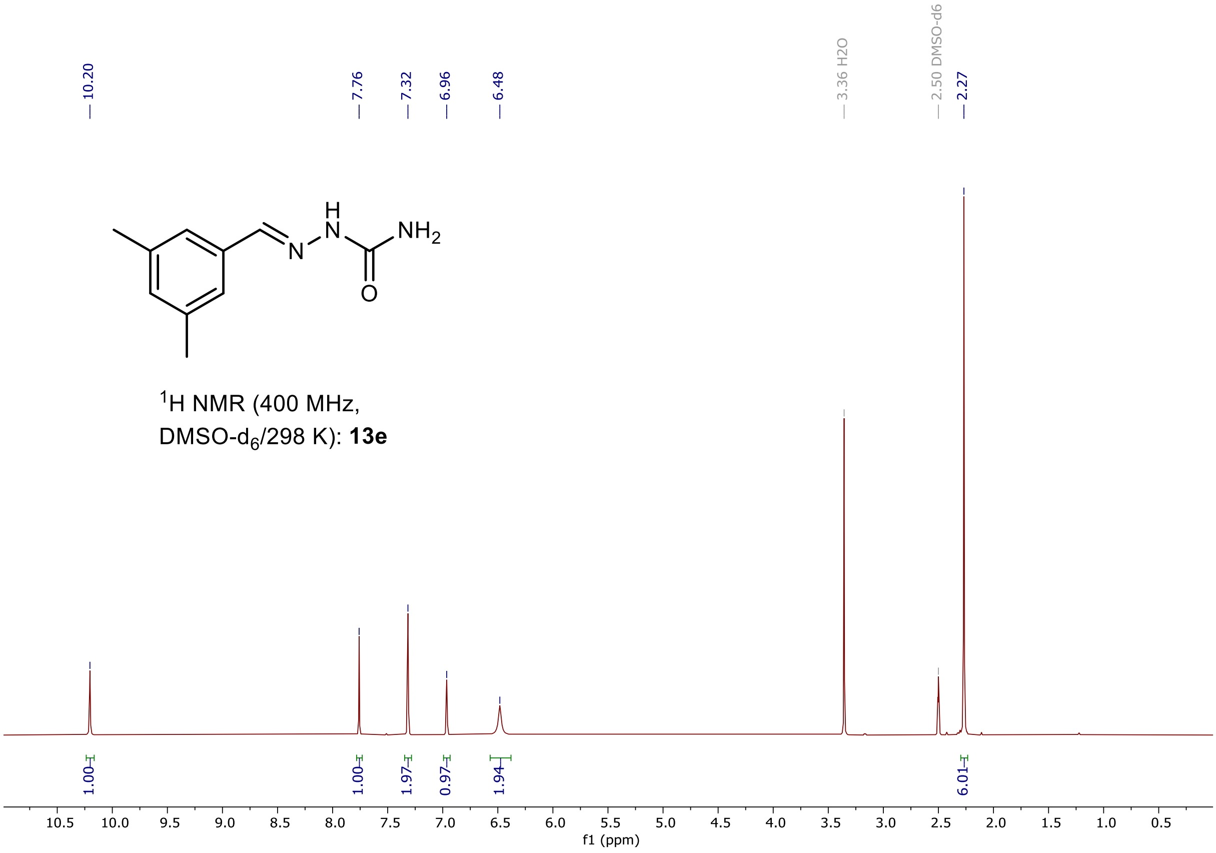

Supplementary Fig. 37: ^{1}H NMR (400 MHz, DMSO-d$_6$, 298 K) of compound **13e**.

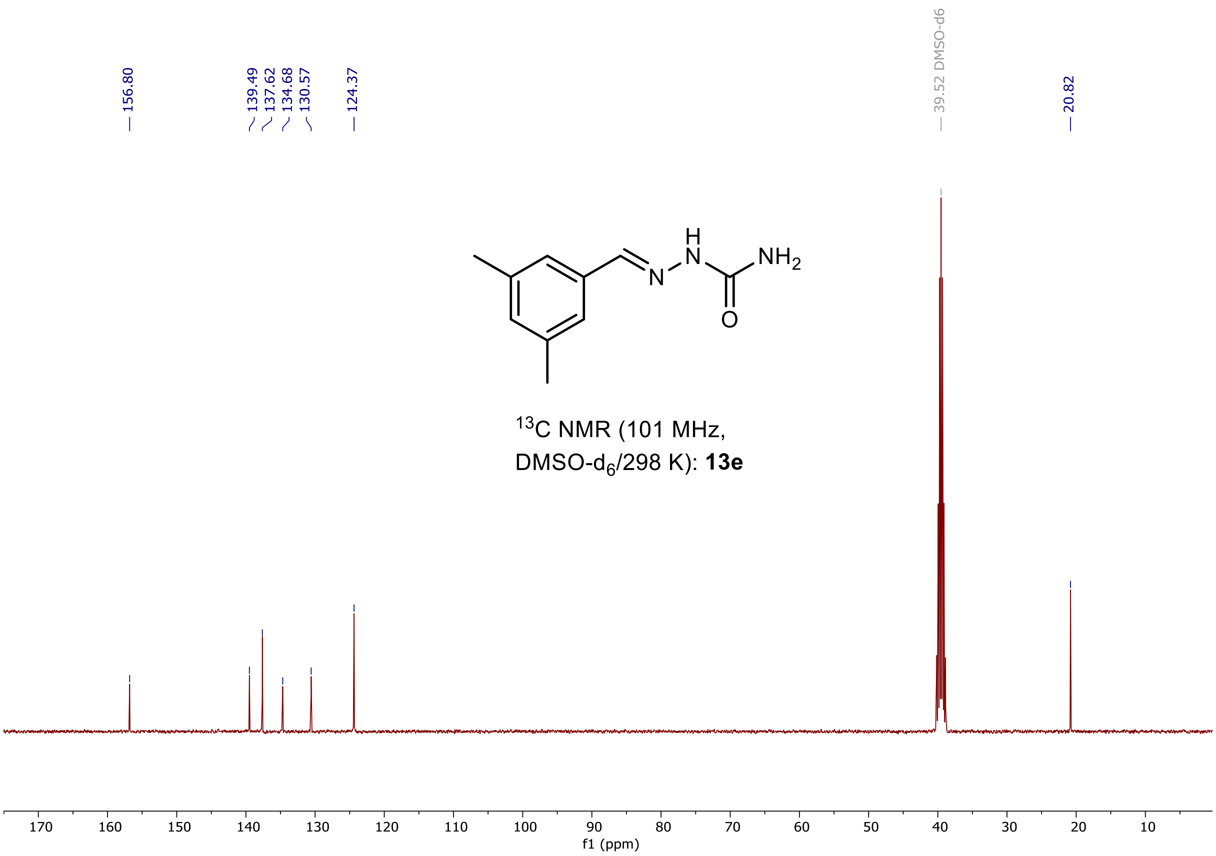

Supplementary Fig. 38: ^{13}C NMR (101 MHz, DMSO-d$_6$, 298 K) of compound **13e**.

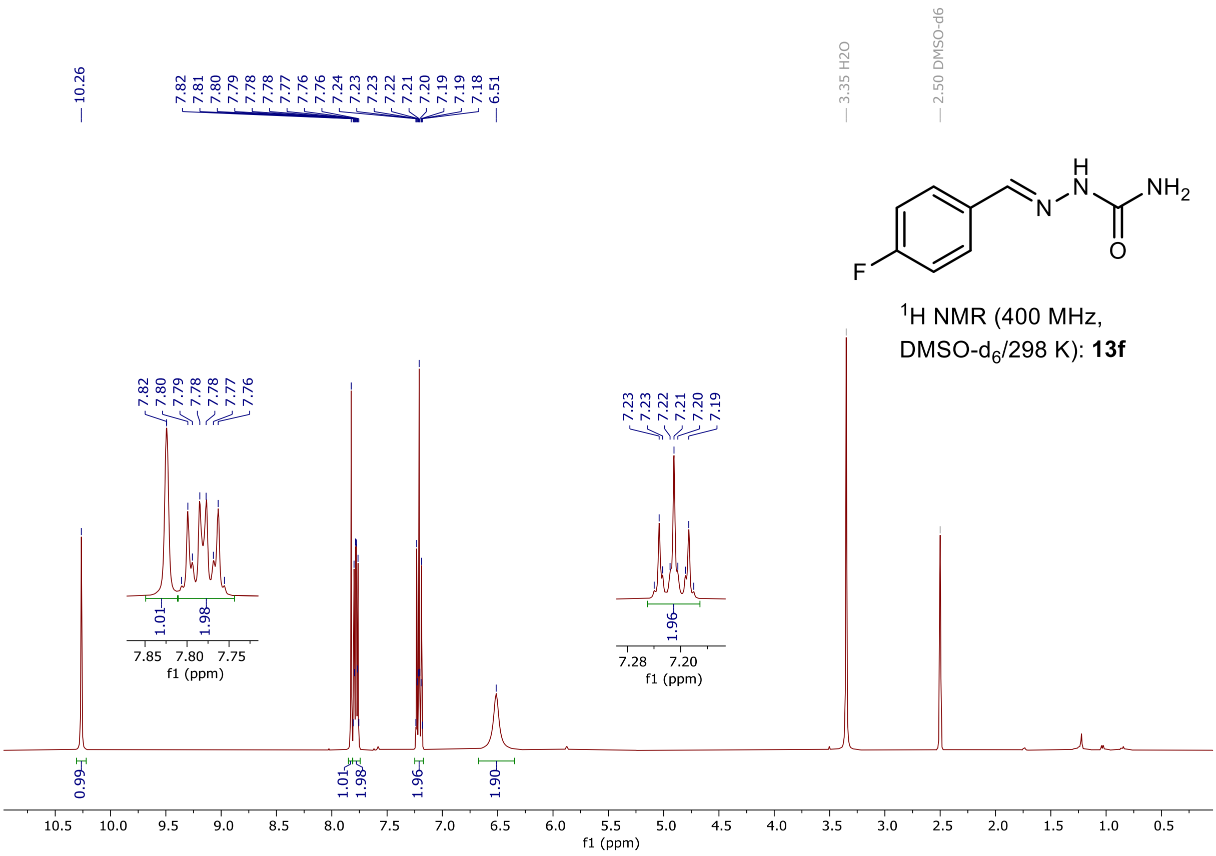

Supplementary Fig. 39: ^{1}H NMR (400 MHz, DMSO-d$_6$, 298 K) of compound **13f**.

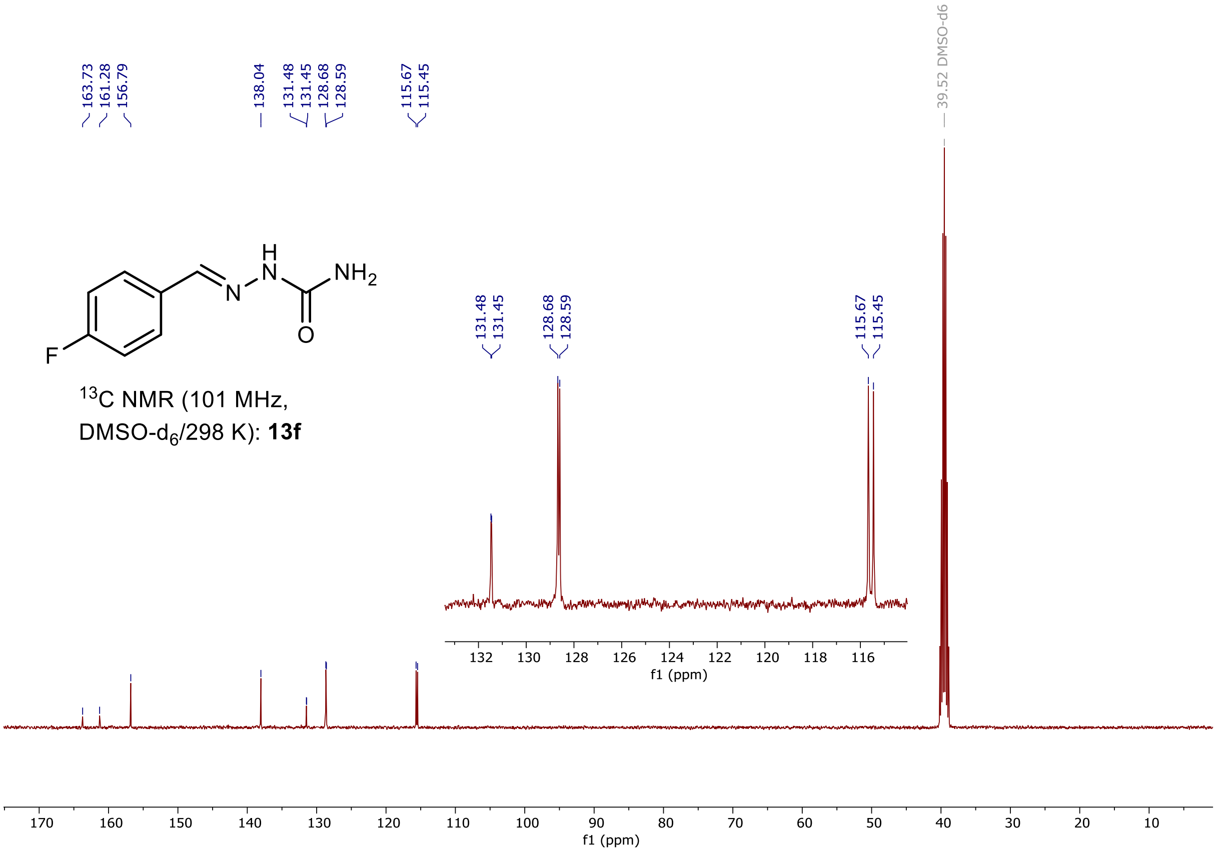

Supplementary Fig. 40: ^{13}C NMR (101 MHz, DMSO-d$_6$, 298 K) of compound **13f**.

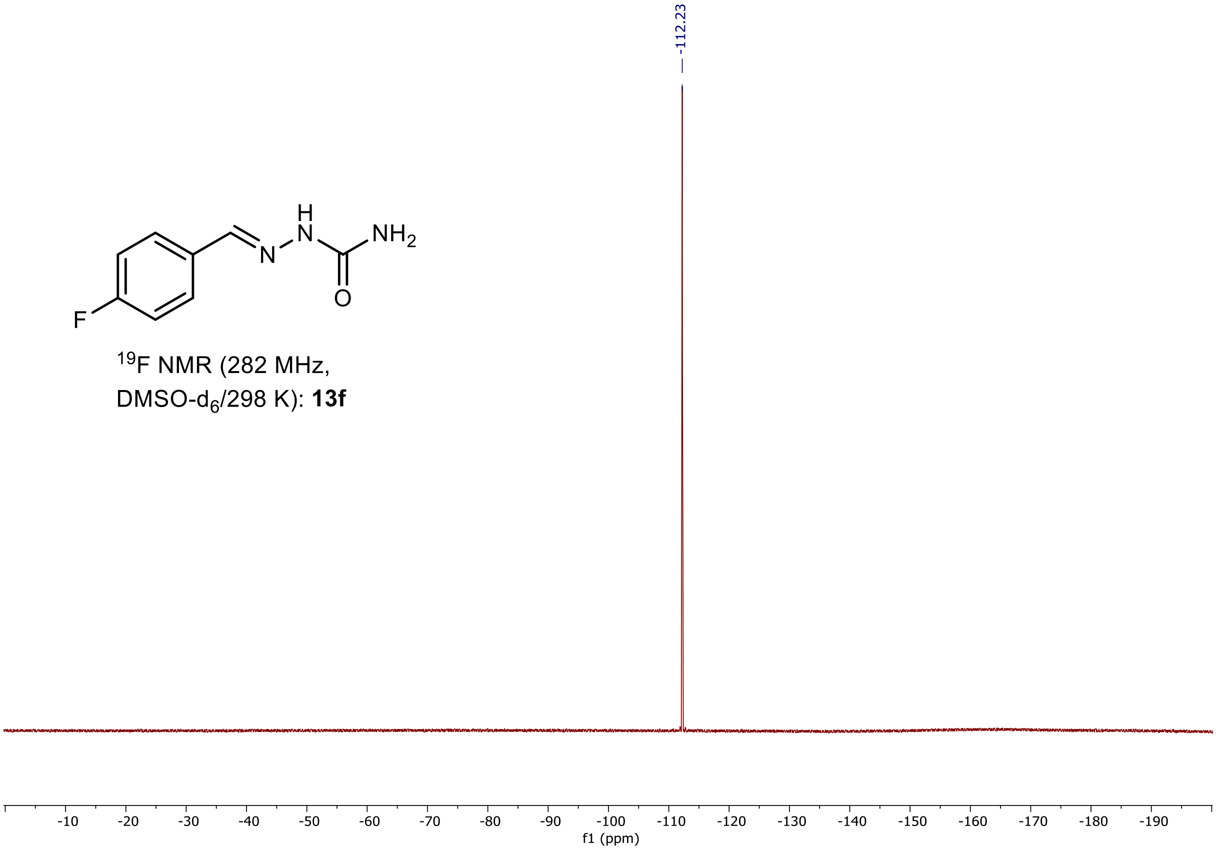

Supplementary Fig. 41: ^{19}F NMR (282 MHz, DMSO-d$_6$, 298 K) of compound **13f**.

5. Supplementary References

1. Harris, R. K., Becker, E. D., Cabral de Menezes, S. M., Goodfellow, R. & Granger, P. NMR nomenclature. Nuclear spin properties and conventions for chemical shifts. *Pure Appl. Chem.* **73**, 1795–1818 (2001).
2. Dörr, M., Waldmann, D. & Waldvogel, S. R. Screening in der Elektrosynthese – Schnelle und nachhaltige Entwicklung der innovativen Chemie von morgen. *GIT Labor-Fachz.* **7–8**, 26–28 (2021).
3. Tellinghuisen, J. Statistical Error Propagation. *J. Phys. Chem. A* **105**, 3917–3921 (2001).
4. Ó'Laoire, C. M. Investigations of oxygen reduction reactions in nonaqueous electrolytes and the lithium-air battery. (Northeastern University, Boston, Massachusetts, 2010, p. 84).
5. Franco, C. & Olmsted III, J. Photochemical determination of the solubility of oxygen in various media. *Talanta* **37**, 905–909 (1990).
6. Achord, J. M. & Hussey, C. L. Determination of dissolved oxygen in nonaqueous electrochemical solvents. *Anal. Chem.* **52**, 601–602 (1980).
7. Heard, D. M. & Lennox, A. J. J. Electrode materials in modern organic electrochemistry. *Angew. Chem. Int. Ed.* **59**, 18866–18884 (2020).
8. Skov, H., Benter, T., Schindler, R. N., Hjorth, J. & Restelli, G. Epoxide formation in the reactions of the nitrate radical with 2,3-dimethyl-2-butene, *cis*- and *trans*-2-butene and isoprene. *Atmos. Environ.* **28**, 1583–1592 (1994).
9. Guo, T., Gao, Y., Li, Z., Liu, J. & Guo, K. Cyclopropenium-activated DMSO for swern-type oxidation. *Synlett* **30**, 329–332 (2019).
10. He, C., Ma, F., Zhang, W. & Tong, R. Reinvestigating $FeBr_3$-catalyzed alcohol oxidation with H_2O_2: Is high-valent iron species (HIS) or reactive brominating species (RBS) responsible for alcohol oxidation? *Org. Lett.* **24**, 3499–3503 (2022).
11. Yang, J., Liu, J., Ge, Y., Huang, W., Neumann, H., Jackstell, R. & Beller, M. Direct and selective synthesis of adipic and other dicarboxylic acids by palladium-catalyzed carbonylation of allylic alcohols. *Angew. Chem. Int. Ed.* **59**, 20394–20398 (2020).
12. Jiang, X., Zhang, J. & Ma, S. Iron catalysis for room-temperature aerobic oxidation of alcohols to carboxylic acids. *J. Am. Chem. Soc.* **138**, 8344–8347 (2016).
13. Hoeschele, J. D., Kasparkova, J., Kostrhunova, H. Novakova, O., Pracharova, J., Pineau, P. & Brabec, V. Synthesis, antiproliferative activity in cancer cells and DNA interaction studies of [Pt(*cis*-1,3-diaminocycloalkane)Cl_2] analogs. *J. Biol. Inorg. Chem.* **25**, 913–924 (2020).
14. Xin, H., Duan, X.-H., Liu, L. & Guo, L.-N. Metal-free, visible-light-induced selective C−C bond cleavage of cycloalkanones with molecular oxygen. *Chem. Eur. J.* **26**, 11690–11694 (2020).
15. Xin, H., Duan, X.-H., Yang, M., Zhang, Y. & Guo, L.-N. Visible light-driven, copper-catalyzed aerobic oxidative cleavage of cycloalkanones. *J. Org. Chem.* **86**, 8263–8273 (2021).
16. Wen, F. & Li., Z. Semicarbazide: A transient directing group for C(sp3)−H arylation of 2-methylbenzaldehydes. *Adv. Synth.Catal.* **362**, 133–138 (2020).
17. Nascimento da Cruz, A. C. et al. Biological evaluation of arylsemicarbazone derivatives as potential anticancer agents. *Pharmaceuticals* **12**, 169 (2019).
18. Wang, Y., Jiang, X. & Wang, B. Cobalt-catalyzed carboxylation of aryl and vinyl chlorides with CO_2. *Chem. Commun.* **56**, 14416–14419 (2020).
